# Large-scale proteomics to identify novel biomarkers linking social determinants of health to heart failure risk

**DOI:** 10.1126/sciadv.aec5063

**Published:** 2026-08-21

**Authors:** Diego Ramonfaur, Rani Zierath, Yimin Yang, Victoria Lamberson, Brian Claggett, Tiffany M. Powell-Wiley, Tammy Leonard, Anna Kucharska-Newton, Keenan A. Walker, Patricia P. Chang, Nrupen Bhavsar, Ganga Bey, Kenneth Butler, Chiadi Ndumele, Josef Coresh, Bing Yu, James S. Floyd, Michelle C. Odden, Amil M. Shah

**Affiliations:** ^1^ Department of Medicine, Brigham and Women’s Hospital, Boston, MA, USA.; ^2^ Department of Internal Medicine, Cleveland Clinic, Cleveland, OH, USA.; ^3^ Penn State College of Medicine, Hershey, PA, USA.; ^4^ University of Texas Southwestern Medical Center and Harold C. Simmons Comprehensive Cancer Center, Dallas, TX, USA.; ^5^ Social Determinants of Obesity and Cardiovascular Risk Laboratory, Cardiovascular Branch, Division of Intramural Research, National Heart, Lung, and Blood Institute, National Institutes of Health, Bethesda, MD, USA.; ^6^ Department of Epidemiology, Gillings School of Global Public Health, University of North Carolina at Chapel Hill, Chapel Hill, NC, USA.; ^7^ Laboratory of Behavioral Neuroscience, National Institute on Aging, Baltimore, MD, USA.; ^8^ Division of Cardiology University of North Carolina at Chapel Hill, Chapel Hill, NC, USA.; ^9^ Division of General Internal Medicine, Department of Medicine, Duke University School of Medicine, Durham, NC.; ^10^ Department of Medicine, University of Mississippi Medical Center, Jackson, MI, USA.; ^11^ Johns Hopkins Bloomberg School of Public Health, Baltimore, MD, USA.; ^12^ NYU Langone Health, New York, NY, USA.; ^13^ University of Texas Health Science Center at Houston School of Public Health, Houston, TX, USA.; ^14^ Departments of Medicine and Epidemiology, University of Washington, Seattle, WA, USA.; ^15^ Department of Epidemiology and Population Health, Stanford University, Stanford, CA, USA.

## Abstract

Adverse social determinants of health (SDOH) associate with greater heart failure (HF) risk. Among 9879 community-based participants of the Atherosclerosis Risk in Communities (ARIC) cohort study, we assessed 4955 plasma proteins using an aptamer-based platform (SomaLogic). We derived an SDOH factor comprising income, education, and area deprivation index (ADI) in Black and white participants. Proteins associated with this factor at Bonferroni significance were tested for associations with incident HF using multivariable Cox proportional hazard models. Among Black participants, 36 proteins were associated with both the SDOH factor and incident HF, 126 were among white participants, and 12 were shared between race strata. Eight of these demonstrated significant mediation effect in causal mediation models. Key results were replicated in 49,396 participants in UK Biobank. Mendelian randomization and colocalization suggested a potentially causal effect of C1q tumor necrosis factor–related protein 1 (C1QTNF1), an adiponectin paralog, on HF. We demonstrate protein biomarkers associated with SDOH burden and HF risk.

## INTRODUCTION

Social determinants of health (SDOH) are defined as the circumstances in which people live and work, and are important drivers for the development of cardiovascular disease (CVD) and associated mortality (*1*, *2*). Among the SDOH, low socioeconomic status (SES) and neighborhood disadvantage are well-documented risk factors for the development of CVD and CVD risk factors (*2*–*7*). Low SES in particular is associated with an increased risk of incident heart failure (HF) and worse prognosis among those with prevalent HF (*8*). Low SES and neighborhood disadvantage are also associated with markers of inflammation, including C-reactive protein (CRP) and interleukin-6 (IL-6) (*9*, *10*), metabolic dysregulation (*11*), kidney dysfunction (*12*, *13*), and neuroendocrine dysfunction (*14*), which together reflect allostatic load—a physiologic response to stressful life experiences and a hypothesized mechanism linking adverse SDOH to CVD (*15*, *16*). However, the specific molecular mechanisms linking adverse SDOH to cardiac dysfunction, and ultimately HF risk, are not well understood.

Minoritized racial and ethnic groups in the United States (US), and Black Americans in particular, are disproportionally burdened by adverse SDOH, including structural racism (*17*). Race is a social construct (*18*), and Black Americans have disparate experiences with the different facets of social and community context including, but not limited to, discrimination (*19*), mass incarceration (*20*), social cohesion (*21*), and voter suppression (*22*) compared to white Americans. Black Americans also have a higher prevalence of CVD (*23*) and greater risk of developing HF compared to other races/ethnicities (*24*). Limited data exist regarding shared and potentially distinct pathways linking SDOH to HF risk in these groups (*25*).

Large-scale proteomic profiling provides a unique opportunity to identify previously unrecognized circulating markers of allostatic load to advance understanding of the biologic linkages between SDOH and HF risk. We leveraged high-throughput aptamer proteomics, prospective HF event ascertainment, and uniform assessments of SES and neighborhood disadvantage among community-based participants in the largely biracial Atherosclerosis Risk in Communities (ARIC) prospective cohort study to identify circulating proteins potentially mediating the association of SDOH with risk of HF among Black and white Americans.

## RESULTS

**Fig. 1.**
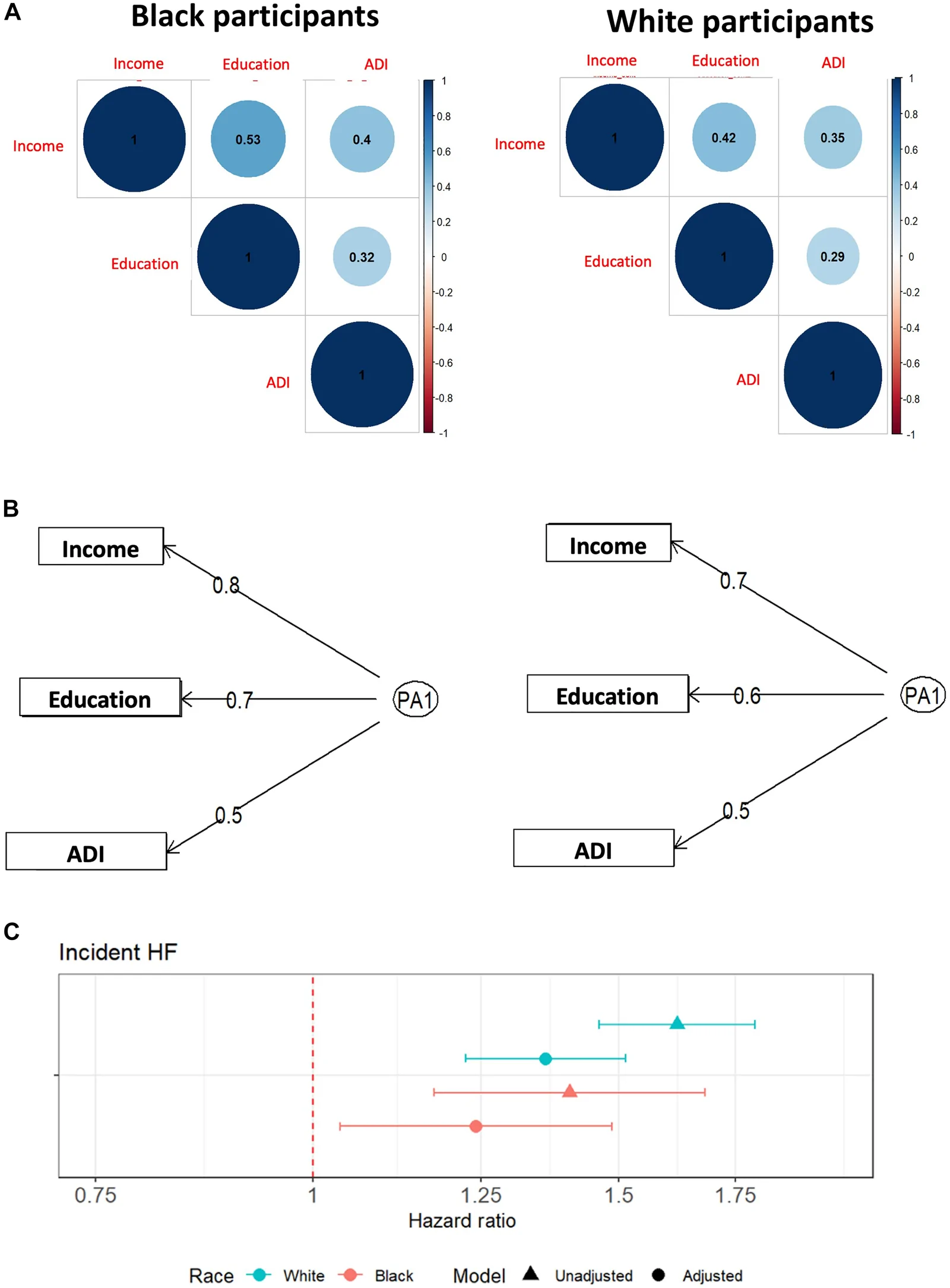
Correlation of SDOH measures and derivation and prognostic relevance of SDOH factor at Visit 3. SDOH measures were coded as higher being worse. (**A**) Spearman correlation matrix among SDOH variables; (**B**) principal axis (PA) exploratory factor loadings; (**C**) association of factor score with risk of incident HF through (year) in bivariate and multivariable Cox proportional hazard models adjusted for age, sex, BMI, eGFR, smoking status, CAD, AF, DM, and hypertension.

The 9879 HF-free participants with proteomics data at Visit 3 (1993–1995) had a mean age of 60 ± 6 years, of which 46% were men, and 21% reported Black race. Black participants were younger, more likely to be female, had a higher prevalence of hypertension and diabetes, and had lower income, lower attained education level, and worse area deprivation index (ADI) rank compared to white participants (Table 1). In each race group, income, education level, and ADI were moderately correlated (Fig. 1A). Using exploratory factor analysis (EFA), each SDOH measure had a factor loading of at least 0.5 for the resulting SDOH factor (Fig. 1B). Over a median follow-up time of 10 years post–Visit 3, 196 of the 1981 Black participants (10%) and 554 of the 7898 white participants (7%) developed HF. In each race group, higher SDOH factor score was significantly associated with risk of incident HF in multivariable Cox proportional hazard models adjusting for age, sex, hypertension, diabetes, smoking status, body mass index (BMI), estimated glomerular filtration rate (eGFR), prevalent coronary heart disease (CHD), and prevalent atrial fibrillation (AF) (Fig. 1C).

**Table 1.** Baseline characteristics of the study population overall and by race group at ARIC Visit 3. Q, quartile.

Variable	Overall	Black race	White race	*P* value
	*n* = 9879	*n* = 1981	*n* = 7898	
Age, mean ± SD, years	60 (6)	59 (6)	60 (6)	<0.0001
Male, *n* (%)	4572 (46)	787 (40)	3785 (48)	<0.0001
BMI, mean ± SD, kg/m^2^	28 (5)	30 (6)	28 (5)	<0.0001
History of smoking, *n* (%)	1730 (18)	430 (22)	1300 (17)	<0.0001
CHD, *n* (%)	632 (6.4)	91 (5)	541 (7)	0.0003
Diabetes, *n* (%)	1731 (18)	546 (28)	1185 (15)	<0.0001
AF, *n* (%)	105 (1.1)	10 (0.5)	95 (1.2)	0.0097
Hypertension, *n* (%)	4407 (45)	1325 (67)	3082 (39)	<0.0001
eGFR, median [Q1, Q3], ml/min/1.73 m^2^	89 [78, 100]	90 [78, 102]	89 [79, 99]	0.0147
Income, *n* (%)				<0.0001
Less than $25,000	3268 (33%)	1221 (62%)	2047 (26%)	
$25,000–$49,999	3539 (36%)	489 (25%)	3050 (39%)	
$50,000–$99,000	2461 (25%)	249 (13%)	2212 (28%)	
Greater than $100,000	611 (6%)	22 (1%)	589 (7%)	
Education, *n* (%)				<0.0001
Below high school graduation	1856 (19%)	682 (34%)	1174 (15%)	
High school graduate	3340 (34%)	436 (22%)	2904 (37%)	
College or vocational school and above	4683 (47%)	863 (44%)	3820 (48%)	
ADI, median rank [Q1, Q3]	38 [28, 53]	78 [61, 88]	34 [27, 44]	<0.0001
Field center, *n* (%)				<0.0001
Forsyth County, NC	2600 (26%)	236 (12%)	2364 (30%)	
Jackson, MI	1714 (17%)	1714 (87%)	0 (0%)	
Minneapolis, MN	2949 (30%)	11 (0.5%)	2938 (37%)	
Washington, MD	2616 (26%)	20 (1%)	2596 (33%)	

### Proteins associated with the SDOH factor and with HF risk

**Fig. 2.**
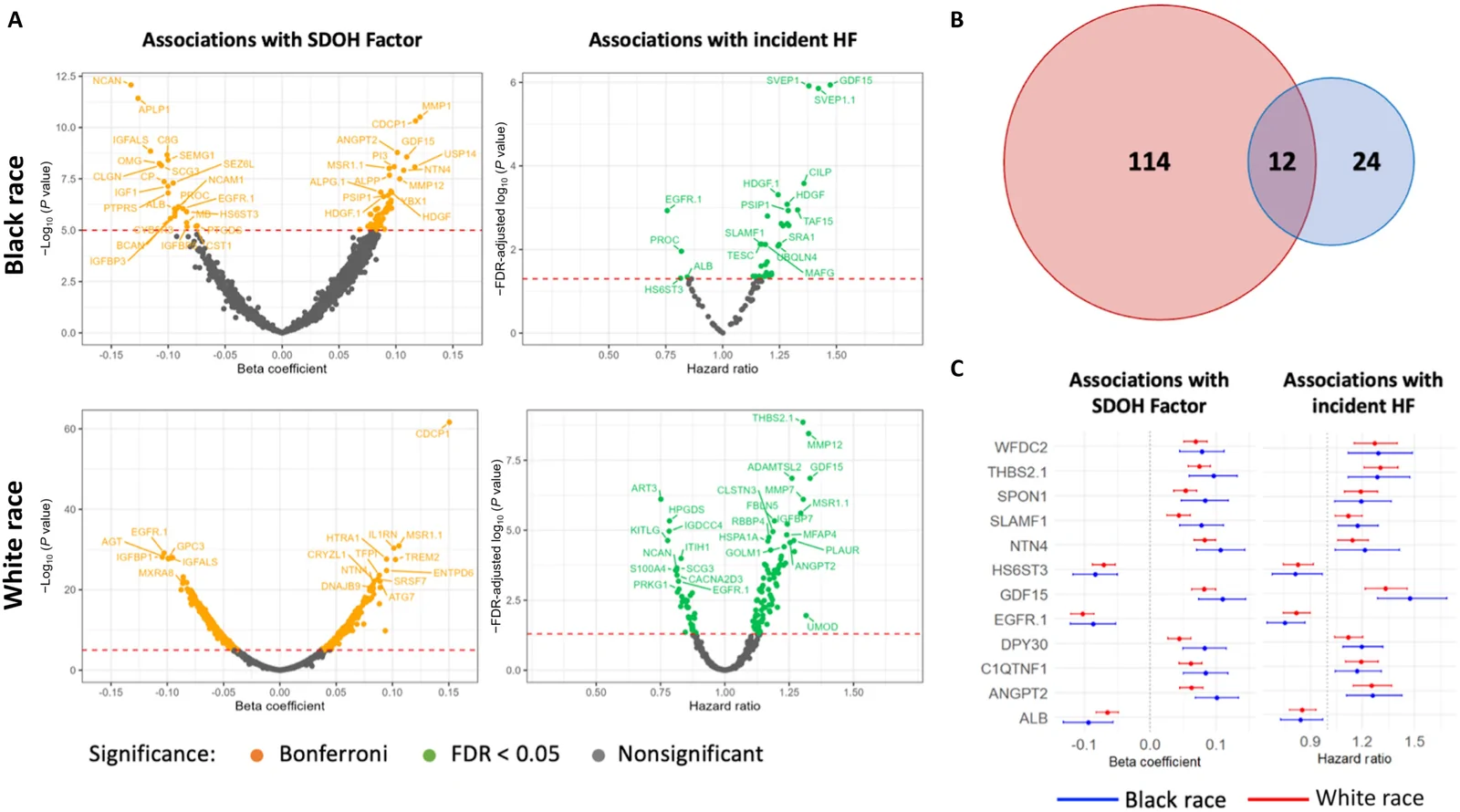
Associations of proteins with SDOH factor and risk of incident HF among Black and white participants. (**A**) Volcano plots of the beta coefficients for cross-sectional associations of proteins with SDOH (left plots) and hazard ratios for associations of SDOH-associated proteins with incident HF (right plots) among Black and white participants. (**B**) Venn diagram showing overlap between SDOH- and HF-associated proteins by race group. (**C**) Forest plot showing effect estimates for associations with SDOH factor and incident HF for the 12 shared SDOH- and HF-associated proteins between race groups.

Among Black participants, 80 of 4955 proteins were cross-sectionally associated with the SDOH factor at Bonferroni level of significance (*P* < 1.0 × 10^−5^) after adjustment for age and sex (Fig. 2A). Of these 80 proteins, 36 were also associated with incident HF in Cox proportional hazard models adjusting for demographics and cardiovascular comorbidities at false discovery rate (FDR) < 0.05 (Fig. 2B). Among white participants, 881 of 4955 proteins were associated with the SDOH factor (Fig. 2C), 126 of which were also associated with incident HF in multivariable Cox proportional hazard models at FDR < 0.05 (Fig. 2C). Twelve SDOH- and HF-associated proteins were shared between Black and white participants. Associations of candidate proteins with SDOH persisted after further adjustment for field center. A sensitivity analysis additionally adjusted for field center yielded similar findings with respect to candidate proteins associated with both the SDOH factor and HF (fig. S2).

### Mediation analysis

**Fig. 3.**
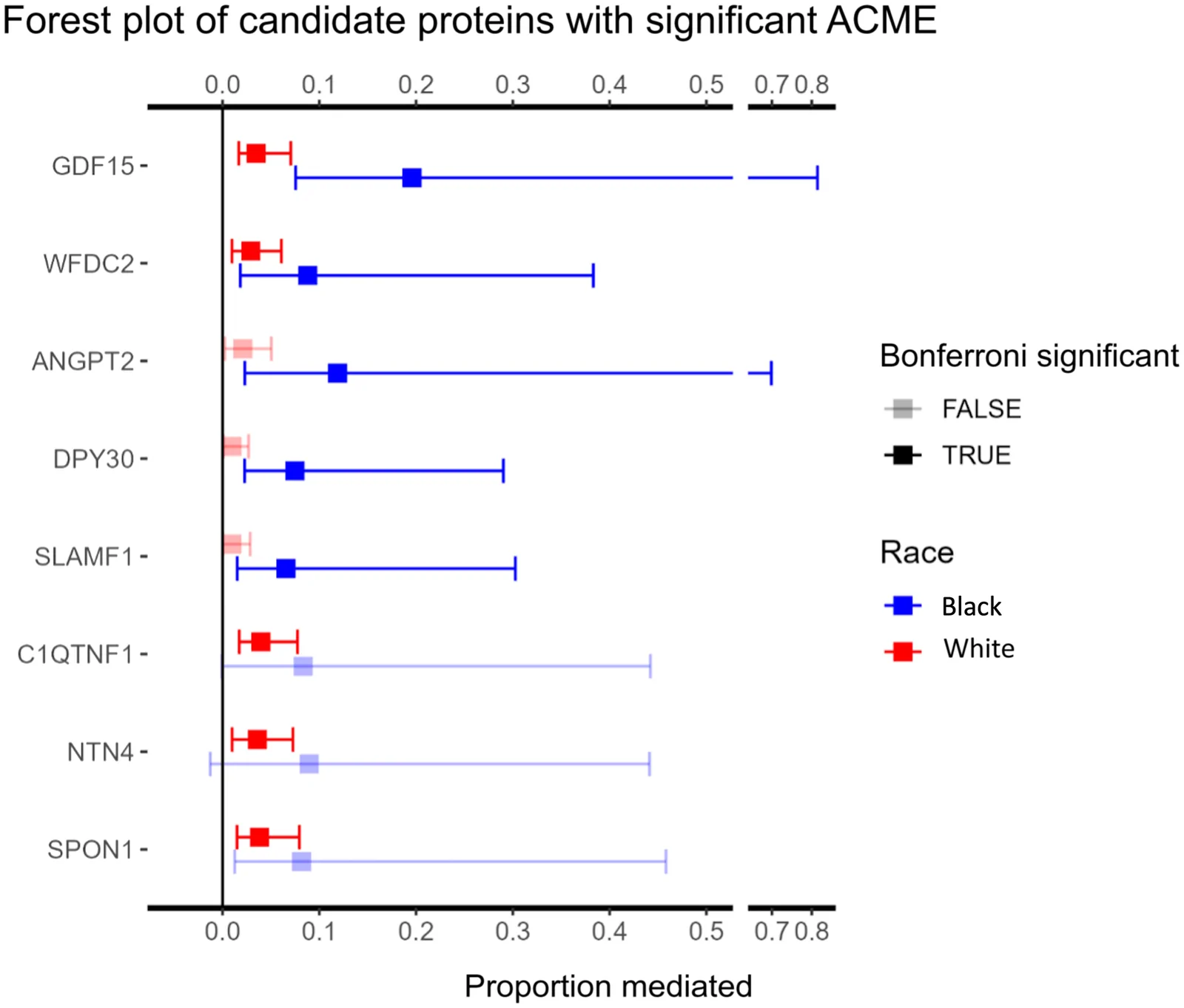
Mediation by candidate proteins of the SDOH factor association with incident HF stratified by race group. Forest plot shows estimated average causal mediation effect (ACME) with associated 95% confidence intervals. Shading indicates statistical significance of mediation effect after Bonferroni correction.

We conducted mediation analysis to explore which of the 12 SDOH- and HF-associated proteins common to both race groups mediated the association between SDOH and HF. Eight proteins were statistically significant mediators of the effect of the SDOH factor on incident HF after Bonferroni correction (*P* = 4.17 × 10^−3^) in at least one race group (Fig. 3): Two proteins were significant mediators in both race groups [growth differentiation factor 15 (GDF15) and whey acidic protein four-disulfide core domain 2 (WFDC2)], three were significant mediators only among Black participants [angiopoietin-2 (ANGPT2), DPY30, and signaling lymphocytic activation moleculte family member 1 (SLAMF1)], and three were significant mediators only among white participants [C1q tumor necrosis factor–related protein 1 (C1QTNF1), netrin 4 (NTN4), and spondin-1 (SPON1)]. Notably, the proportion of the total effect mediated by the candidate proteins tended to be higher among Black compared to white participants (20 versus 3%, respectively, for GDF15; 9 versus 3%, respectively, for WFDC2).

### Mendelian randomization and colocalization

**Fig. 4.**
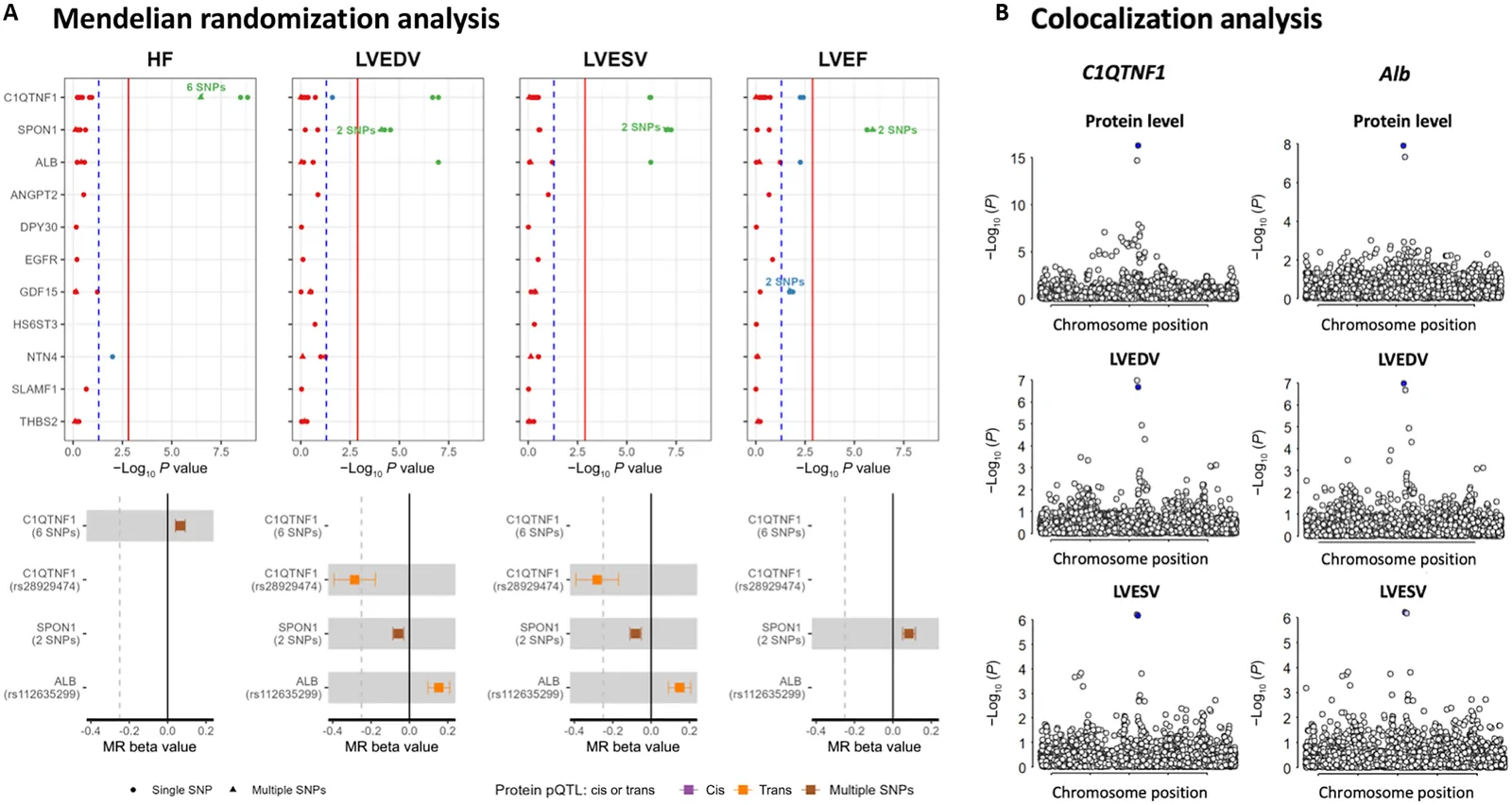
Results of proteogenomic analyses to assess for potential causal associations of proteins with cardiac outcomes. (**A**) MR. Top: MR *P* values for causal effect of protein levels on HF and cardiac structure and function. Hatched line indicates nominal significance (*P* < 0.05), red line indicates significance after Bonferroni multiple testing correction for number of pQTLs tested. Red pQTLs, nonsignificant; blue, nominally significant; green, significant after multiple testing correction. Bottom: Forest plots for significant MR instrumental variables of direction and magnitude of effect of genetically higher protein levels on each outcome. Orange, trans pQTL; brown, multi-pQTL instrumental variable (IV) comprising both cis and trans SNPs. (**B**) Results of colocalization analyses for significant MR associations based on single-SNP trans instruments.

For the 12 candidate proteins, two-sample Mendelian randomization (MR) was used to assess potential causal effects of those proteins on HF and cardiac function. A multi–single-nucleotide polymorphism (SNP) instrument for complement C1QTNF1 demonstrated MR association with HF at Bonferroni-corrected level of significance (Fig. 4A). In addition, a trans protein quantitative trait locus (pQTL) for C1QTNF1 demonstrated potential causal associations with smaller left ventricular end diastolic volume (LVEDV) and smaller left ventricular end systolic volume (LVESV), which were confirmed with colocalization analysis (Fig. 4B). Notably, data from the Genotype-Tissue Expression (GTEx) project demonstrate that C1QTNF1 is differentially expressed in heart, arteries, and adipose tissue (fig. S3). Significant MR associations were also observed for a multi-SNP instrument for SPON1 with smaller LVEDV and LVESV and higher left ventricular ejection fraction, and for trans pQTLs for albumin with larger LVEDV and LVESV. We additionally observed nominally significant effects of both C1QTNF1 and SPON1 on magnitude of myocardial interstitial fibrosis (fig. S4).

### UK Biobank replication analysis

Among 49,396 UK Biobank (UKB) participants at the baseline assessment, educational attainment and Townsend Deprivation Index (TDI) were moderately correlated with household income, whereas education was weakly correlated with TDI (fig. S5A). Using EFA, each SDOH measure had a factor loading of 0.2 or more for the resulting factor (fig. S5B). Over a median follow-up time of 13.6 [12.8, 14.3] years, 2242 (5%) of participants developed HF. A higher SDOH factor score was associated with a greater risk of incident HF in Cox proportional hazard models adjusted for demographics and cardiovascular comorbidities (fig. S5C).

**Fig. 5.**
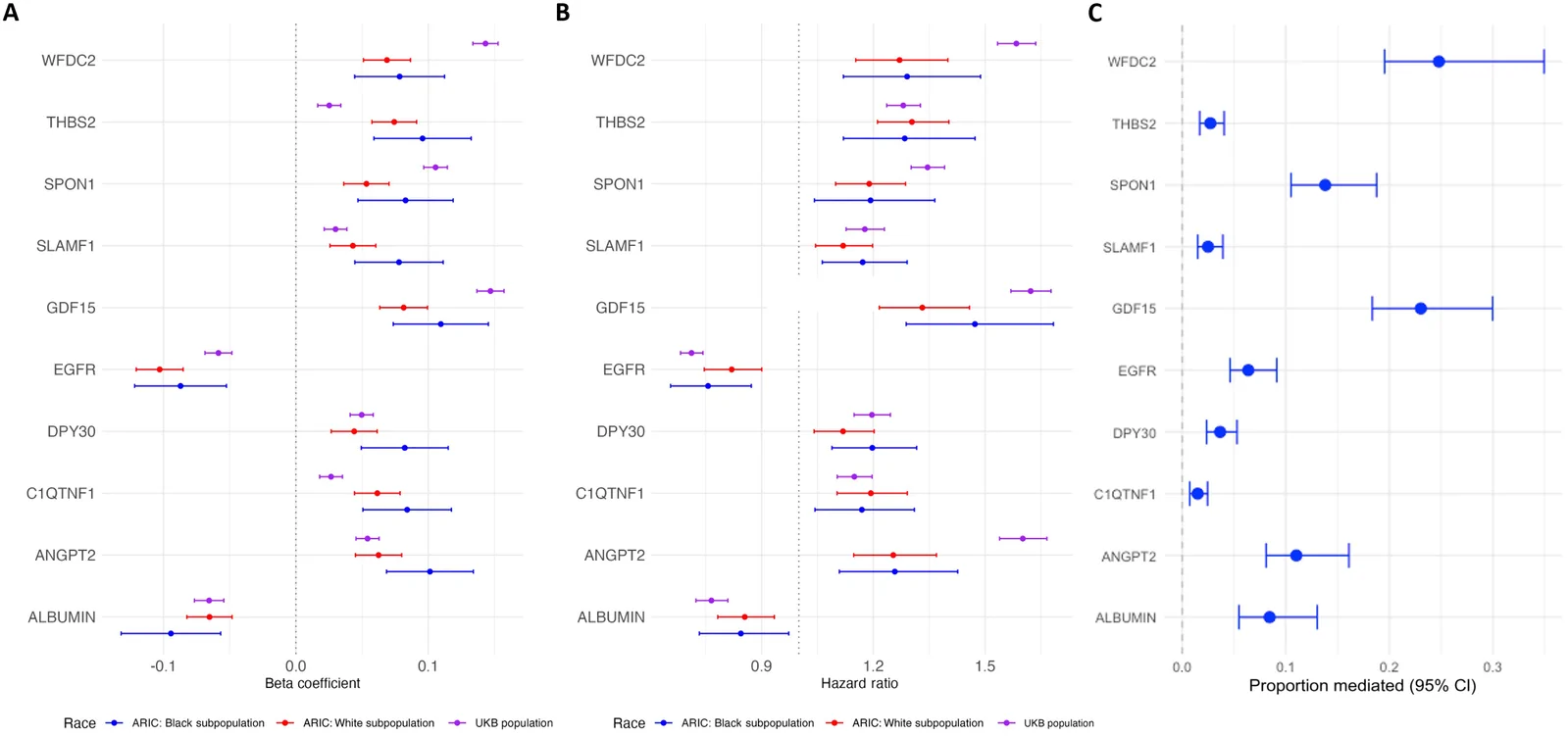
Associations of UKB proteins with SDOH factor score and HF. (**A**) Forest plot showing effect estimates for associations with SDOH factor for the 10 available candidate proteins. (**B**) Forest plot showing effect estimates for associations between the available 10 candidate proteins and incident HF. (**C**) Forest plot demonstrating mediation by candidate proteins of the SDOH factor association with incident HF. CI, confidence interval.

Ten of the 12 candidate proteins identified in the ARIC analysis were assessed in the UKB, with nine using the Olink platform, which demonstrated moderate correlation with the candidate proteins identified in ARIC using the SomaLogic platform (fig. S6). All 10 proteins were cross-sectionally associated with the SDOH latent factor score at a Bonferroni level of significance (*P* < 1.0 × 10^−5^) (Fig. 5A). As in ARIC, higher levels of eight of the 10 candidate proteins were associated with a greater risk of incident HF in Cox proportional hazard models adjusted for demographics and comorbidities, while higher levels of epidermal growth factor receptor (EGFR) and albumin were associated with a lower risk of incident HF Fig. 5B). In mediation analysis, each of the 10 available candidate proteins demonstrated a significant mediation effect of the SDOH factor on incident HF with Bonferroni correction (Fig. 5C).

## DISCUSSION

Using large-scale proteomics, standardized assessments of SES and neighborhood context, and prospectively ascertained incident HF events in a large biracial community–based cohort, we identified 12 proteins independently associated with SDOH burden and with incident HF among both Black and white participants. Of these 12 proteins, MR identified a potentially causal effect of C1QTNF1, an adiponectin paralog associated with inflammation, on HF. Furthermore, causal mediation analysis identified nine proteins as statistically significant potential mediators of the association between the SDOH factor and incident HF in at least one race group, two of which (WFDC2 and GDF15) were significant in both race groups. Cross-platform replication of these findings was demonstrated in the UKB. This study demonstrates the potential for large-scale proteomics to find previously unrecognized biomarkers linking SDOH to HF.

Low SES and neighborhood disadvantage are well-documented risk factors for the development of cardiovascular risk factors and CVD (*1*, *3*–*7*). Low SES, in particular, is associated with an increased risk of incident HF and worse prognosis among those with prevalent HF (*8*). Adverse SDOH, including low SES and neighborhood disadvantage, are associated with markers of inflammation, including CRP and IL-6 (*9*–*11*, *14*); metabolic dysregulation, such as homocysteine, cholesterol, eGFR, and albuminuria (*10*–*15*); and neuroendocrine dysfunction, such as cortisol and norepinephrine (*14*, *15*). Together, these biomarkers are common measurements of allostatic load—a physiologic response to stressful life experiences and a hypothesized mechanism linking SDOH to CVD (*15*, *16*). Despite these associations, the pathophysiologic mechanisms linking SDOH to cardiac dysfunction and HF risk remain incompletely understood. Using large-scale proteomics, we have identified numerous circulating biomarkers linking SDOH to HF risk.

Black Americans are disproportionally burdened by adverse SDOH, including structural racism and discrimination (*19*). Compared to white Americans, Black Americans have different and often unjust experiences with the various facets of social and community context (*10*–*13*). Moreover, Black Americans are disproportionately burdened by historical redlining policies, biased housing and lending practices, and environmental exposures including air pollution, which have been associated with greater HF risk (*26*). Given the important role of race as a social construct in conditioning an individual’s exposure to and lived experience of a range of adverse SDOH, we stratified our analysis by self-reported race to ensure we identified generalizable and robust SDOH-protein–incident HF associations; we identified 12 plasma proteins with consistent associations with SDOH burden and HF risk in Black and white participants. These proteins together implicate inflammation, cellular proliferation, extracellular matrix composition, and tissue remodeling. Of the 10 of these assessed in the UKB, all demonstrated consistent associations with SDOH burden and HF risk despite being assessed using a different proteomic platform with a distinct affinity-based capture technology, highlighting the potential generalizability of these results.

Our study demonstrates an association between C1QTNF1 and adverse SDOH, in addition to risk of developing HF. C1QTNF1, also known as C1q/TNF-related protein-1 (CTRP1), is an adiponectin paralog that plays a role in the regulation of metabolism and inflammation (*27*). CTRPs are dysregulated in metabolic syndrome (*28*, *29*), diabetes (*30*), coronary artery disease (CAD) (*31*, *32*), and nonalcoholic fatty liver disease (*33*). C1QTNF1is highly expressed in lipid-laden foam cells and is linked to local inflammation. High plasma levels of C1QTNF1 in particular have been associated cross-sectionally with HF (*34*), possibly as a result of activation of myocardial fibrosis pathways (*35*). Furthermore, C1QTNF1 may worsen cardiac fibrosis in the presence of catecholaminergic stimuli (*35*), which can be secondary to acute or chronic stress (*36*). The results of MR and colocalization analyses further support a causal effect of C1QTNF1 on HF, subclinical alterations in left ventricle (LV) structure and function, and LV myocardial interstitial fibrosis. That C1QTNF1-specific antibodies have been shown to block the secretion of proatherosclerotic mediators underscores its potential as a therapeutic target (*37*). MR analysis also supported a potential causal association of SPON1, another SDOH- and HF-associated protein, with LV size and function. SPON1 is a matricellular protein primarily implicated in dementia and axonal development (*38*). However, it is expressed in the LV and left atrium, and the relevant SPON1 protein quantitative trait locus (pQTL)s are also expression quantitative trait locus (eQTL)s in these tissues. Animal models implicate SPON1 in genetically mediated hypertension and in cardiac remodeling that results from ischemia-reperfusion (*39*, *40*).

Causal mediation analysis identified GDF15 and WFDC2 as significant potential mediators of the SDOH-HF association in both race groups. GDF15, a member of the transforming growth factor beta family, is an established biomarker of HF risk (*41*–*43*) and cardiac dysfunction (*44*). WFDC2, also known as circulating human epididymis protein 4 (HE4), is an established biomarker for epithelial ovarian cancer (*45*). However, circulating WFDC2 levels are also associated with risk of incident HF and adverse outcomes in prevalent HF (*46*, *47*), potentially related to fibrosis given its sequence similarities with proteinase inhibitors (*48*). Additional previously unrecognized SDOH- and HF-associated proteins found in this analysis include SLAMF1, which has a role in immune regulation (*49*), and ANGPT2, which is involved in both angiogenesis and innate immunity and has previously been associated with HF risk (*50*). EGFR is a member of the ErbB family receptors, and the EGFR tyrosine kinase inhibitor osimertinib has been previously associated with cardiac dysfunction and HF (*51*). The neuregulin-1 (NRG-1)/ErbB signaling pathway has been implicated in cardiovascular homeostasis through regulation of cardiac autonomic balance (*52*).

Our findings should be interpreted in the context of potential limitations. Ascertainment of income and educational attainment was self-reported, which may result in misclassification. Furthermore, social adversity is a complex construct that is likely influenced by more unmeasured factors. While our SDOH factor was based on measures of SES and neighborhood socioeconomic context, additional key dimensions of SDOH including discrimination/racism, built environment, historical redlining policies, environmental exposures, health care access, and psychosocial factors were not considered. The size of the white race stratum was larger than the Black race stratum, resulting in lower power in the Black stratum compared to the white stratum for associations of plasma proteomics with both the SDOH factor and with incident HF. While high throughput aptamer-affinity proteomics enables broad sampling of the proteome, target protein specificity is variable across aptamers. Therefore, further studies using targeted assays are necessary to validate our findings. This study was observational, and therefore, unmeasured confounding may affect our estimates and preclude causal inference. MR and colocalization analyses help mitigate, but cannot eliminate, this concern for key protein-HF outcomes. Furthermore, the instrumental variables (Ivs) for MR analyses were derived from studies of European ancestry individuals given the limited data available from African ancestry individuals. However, while lack of diverse ancestry may limit our ability to identify functionally important pQTLs for MR analysis (*53*), we do not expect it to affect the validity of the identified MR associations. In addition, causal mediation analysis assumes the absence of unmeasured confounders. While this assumption is not testable and likely violated, this analysis does allow us to estimate the magnitude of the SDOH-HF association statistically accounted for by the proteins of interest. Within the ARIC study, we identified proteins reproducibly associated with SDOH burden and HF risk across Black and white participants. We believe that this consistency speaks to the generalizability of these associations, as both SDOH burden and HF risk differ by race especially within the US, and Black participants are overwhelmingly geographically distinct from white participants in ARIC due to study design. Differences in SDOH were less pronounced between Black and white participants of the UKB, so the replication of our findings in this cohort from a different country and using a different proteomics platform reinforces their validity. However, longitudinal study participants tend to be healthier, have higher SES, and have more access to health care than the general population, which may limit broad generalizability.

To conclude, large-scale plasma proteomics identified 12 proteomic biomarkers associated with SDOH burden and risk of incident HF consistently in both Black and white adults with key findings replicated in a geographically independent cohort using a distinct proteomics platform, nine of which significantly mediated the relationship between SDOH and incident HF in at least one group. MR and colocalization analysis identified a previously unrecognzied, potentially causal effect of C1QTNF1, an adiponectin paralog associated with inflammation, on HF and LV structure.

## MATERIALS AND METHODS

### Study population

The design and procedures of the ARIC study have been previously described (*54*). ARIC is a prospective epidemiologic study that initially enrolled 15,792 participants aged 45 to 64 years between 1987 and 1989 in four US communities using probability sampling: Forsyth County, North Carolina; Jackson, Mississippi; suburbs of Minneapolis, Minnesota; and Washington County, Maryland (Table 1). The field centers represent geographically distinct regions within the US, and the racial composition of the study population varied such that the study population recruited from Jackson, Mississippi was entirely self-identified Black or African American race. This study was approved by the institutional review board (IRB) at each study center (IRB approval nos. IRB00311999 and IRB00311861). All participants provided written informed consent. This analysis primarily included participants attending Visit 3 (1993–1995), when plasma proteomics and SDOH variables were assessed. Among 13,688 participants who were alive at the time of Visit 3, 12,887 attended the study visit, of whom 9879 were included in this analysis after excluding those with missing proteomic data (*n* = 1409), with prevalent HF (*n* = 898), reporting non-Black and non-white race (*n* = 38), or missing SES variables (*n* = 903).

The study protocol was approved by IRB at each field center. All participants provided written informed consent. Anonymized data from the ARIC study are available at the National Heart, Lung, and Blood Institute (NHLBI) Biologic Specimen and Data Repository Information Coordinating Center and can be accessed through the website (https://biolincc.nhlbi.nih.gov/studies/aric/). Requests for access of ARIC data may also be submitted to the ARIC Publications Committee according to established study procedures. The code and statistical packages used for analyses in this study are available from the corresponding author upon reasonable request.

### Protein measurement

Plasma protein levels were measured by SomaLogic using the Multiplexed Slow Off-Rate Modified Aptamer assay (SomaScan v4) on plasma samples collected and stored at −80°C at ARIC Visit 3. A total of 4955 aptamers measuring levels of 4712 unique proteins were present for analysis after quality control (*55*). Protein levels were standardized to a mean of 0 and standard deviation of 1. Detailed descriptions of protein measurement, data standardization, and quality control procedures have been previously published and are provided in the Supplementary Methods.

### Measurement of SES variables

Education was assessed at Visit 1 by asking participants for their highest attained level of education with seven categories: <8th grade, 8th to 11th grade, high school or equivalent diploma, any level of vocational school, 1 to 4 years of college, ≥4 years of college, and any graduate or professional education (*56*). In this analysis, education was modeled as a five-level ordinal variable, with vocational school, 1 to 4 years of college, and ≥4 years of college categories combined into one category. At Visit 3, ARIC study participants were asked to classify their total family income into one of the following nine categories: <$5000; $5000–$7999; $8000–$11,999; $12,000–$15,999; $16,000–$24,999; $25,000–$34,999; $35,000–$49,000; $50,000–$74,999; and ≥$75,000. Neighborhood ADI, a multiparametric measure of neighborhood-level socioeconomic deprivation, was assessed at Visit 3 using the method of Singh *et al.* (*57*, *58*). The ADI incorporates metrics of educational and occupational status, income and employment distributions, and housing conditions (see Supplementary Methods). ARIC study participants’ addresses were geocoded to calculate individual ADI and expressed as a national percentile rank ranging from 1 to 100. The distribution of the SES variables is displayed in fig. S1. The Supplementary Methods show details regarding the assessment of demographics and clinical characteristics including cardiovascular risk factors and disease in ARIC.

### HF ascertainment

HF was ascertained through active surveillance of hospital discharges and annual follow-up calls to participants to identify hospitalizations’ HF-associated International Classification of Diseases (ICD) codes. For Visit 3 analyses, HF hospitalization was defined as a hospitalization with an ICD Ninth Revision code of 428 as previously described (*59*). HF was ascertained through 10 years post–Visit 3 (31 December 2003). Prevalent HF was defined based on an incident HF event occurring before the Visit 3 date.

### Statistical analysis

**Fig. 6.**
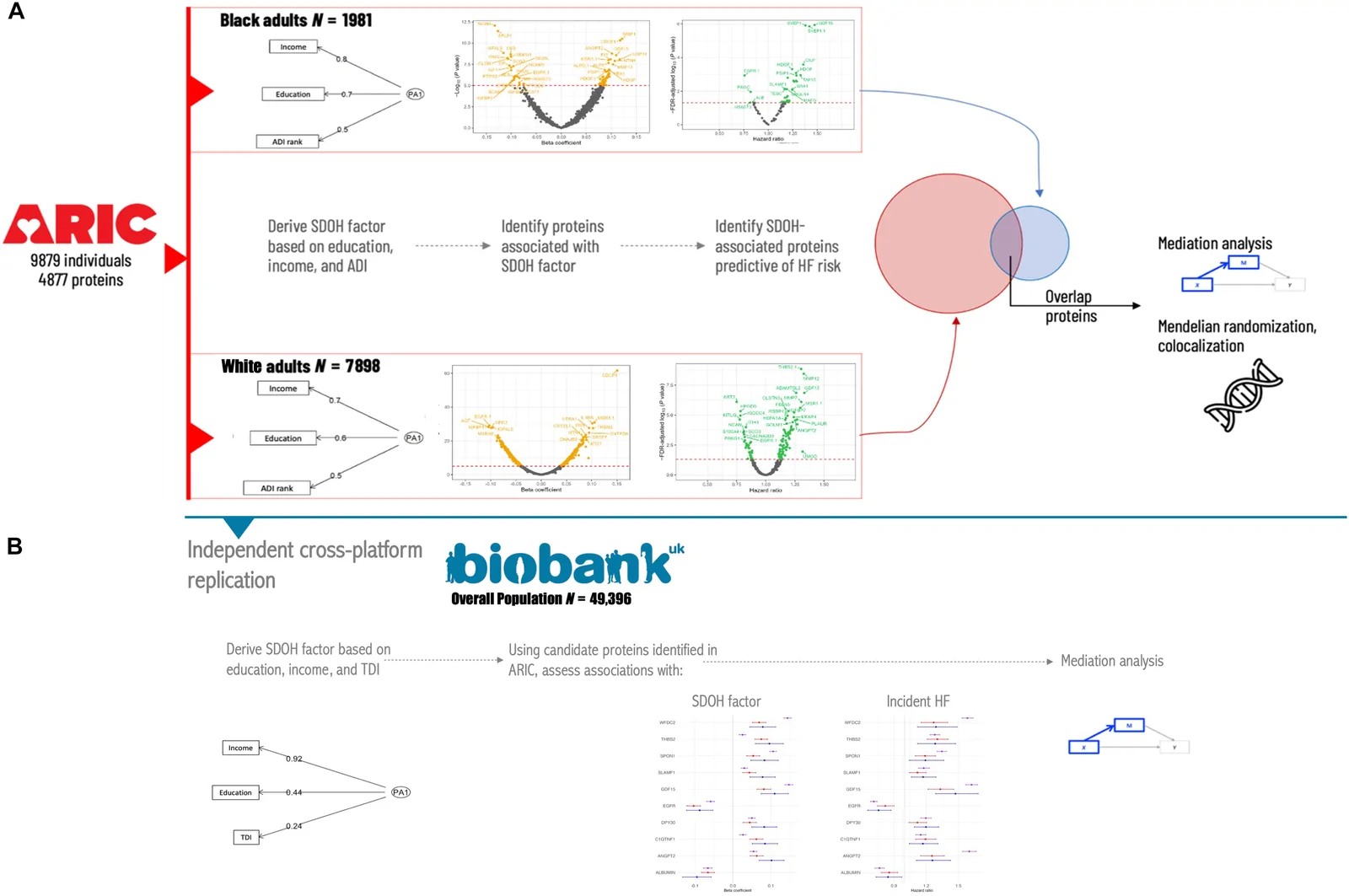
Study design. (**A**) Study design of the analysis in ARIC. (**B**) Study design of the replication analysis in UKB.

Given the important differences in SES and experiences of social and community context by race, and to enable identification of protein associations consistent across this heterogeneity, all analyses were stratified by self-reported race: Black race and white race (fig. S1 and Fig. 6).

#### Association of proteins with the SDOH factor and with incident HF

Within each self-reported race group, we performed EFA to derive an SDOH factor based on education, income, and ADI assessed at Visit 3, as detailed in the Supplementary Methods (*60*). Within each self-reported race group, we identified proteins assessed at Visit 3 that were cross-sectionally associated with the Visit 3 SDOH factor using multivariable linear regression models adjusting for age and sex at a Bonferroni-corrected level of significance (*P* = 1.0 × 10^−5^). Among proteins significantly associated with the SDOH factor in each group, we then assessed their association with incident HF over 10 years post–Visit 3 using multivariable Cox proportional hazard regression models and a Benjamini-Hochberg FDR significance (*q* < 0.05) in each race group. Models were adjusted for age, sex, hypertension, diabetes, smoking status, BMI, eGFR, prevalent CHD, and prevalent AF. The resulting proteins comprised our “SDOH- and HF-associated proteins.” Last, we identified “SDOH- and HF-associated proteins” that were shared between both race groups, and these comprised our final set of candidate proteins for additional analyses. We performed a sensitivity analysis further adjusting for field center in assessing the associations of candidate proteins with the SDOH factor and with incident HF to account for potential heterogeneity by study field center.

#### Mediation analysis

Within each race group, we investigated the final candidate proteins assessed at Visit 3 as potential mediators of SDOH factor (exposure) on incident HF post–Visit 3 (outcome) using causal mediation analysis with the R package “mediation” (v4.5.0) and as detailed in the Supplementary Methods (*61*). Models were adjusted for age, sex, BMI, eGFR, smoking, CAD, diabetes, AF, and hypertension. Proteins with significant average causal mediation effect (ACME) after Bonferroni correction based on the number of proteins tested were subsequently identified among each race group.

#### MR and colocalization

We performed two-sample MR (*62*) to assess the potential causal effect of our final candidate proteins on incident HF and cardiac function. For exposure and outcome data in each analysis, we used genetic summary statistics from two separate groups of European ancestry to reduce bias. Ivs for pQTLs were drawn from the INTERVAL (*63*), AGES (*64*), and Fenland studies (*27*). Instruments were analyzed individually. We performed colocalization analysis, assuming a single causal variant, on significant MR hits located in a single genomic region. We additionally performed tissue expression analysis using data from the GTEx platform. Please refer to the Supplementary Methods for a detailed description of MR, colocalization, and tissue expression analyses.

### Independent replication cohort: UKB

The UKB is a large, prospective cohort study of over 500,000 individuals aged 40 to 69 years who were recruited between 2006 and 2010 for the baseline assessment (*65*). Proteomic profiling was performed on the blood samples of 54,000 participants using the Olink Explore assay, capturing almost 3000 unique proteins (*66*). A total of 9396 participants with proteomic and social determinant of health data and of Black or white race were included in the replication analysis. Household income and educational attainment were self-reported, and TDI was used as measure of area deprivation (see the Supplementary Materials) (*67*–*71*). Due to the similar distribution of income, TDI, and education levels between white and Black UKB participants, the replication analysis was not stratified by race group.

The EFA analysis, as previously described, was performed to create a latent factor derived from household income, educational attainment, and TDI. Ten of the 12 candidate proteins identified in the ARIC were also available in the UKB, nine from Olink assay, including epidermal growth factor receptor (EGFR), albumin, WFDC2, thrombospondin-2 (THBS2), SPON1, SLAMF1, GDF15, DPY30, C1QTNF1, and ANGPT2. The associations between factor score and candidate proteins were assessed with linear regression, and associations of SDOH-associated proteins with incident HF were assessed with Cox proportional hazard. Last, mediation analysis was performed, and proteins with significant ACME after Bonferroni correction were identified (Fig. 6). All models were adjusted for demographics and comorbidities with Bonferroni correction as above.

## Supplementary Materials

### This PDF file includes:

Supplementary Methods Figs. S1 to S6 References
