# Supplementary material for: Large-scale proteomics to identify novel biomarkers linking social determinants of health to heart failure risk
Source: Sci Adv. 2026 Aug 21;12(34):eaec5063. doi: 10.1126/sciadv.aec5063 (PMC13496179; doi:10.1126/sciadv.aec5063)
Supplement: Supplementary file 1 [file sciadv.aec5063_sm.pdf]

Science Advances
AAAS

Supplementary Materials for

Large-scale proteomics to identify novel biomarkers linking social determinants of health to heart failure risk

Diego Ramonfaur *et al.*

Corresponding author: Amil M. Shah, amil.shah@utsouthwestern.edu

Sci. Adv. **12**, eaec5063 (2026)
DOI: 10.1126/sciadv.aec5063

This PDF file includes:

Supplementary Methods
Figs. S1 to S6
References

Supplemental Methods

Protein Measurement

The Olink Explore 3072 assay was also performed in plasma samples from 110 participants at Visit 5 in which the SomaLogic v4.1 was also performed. Given the larger sample size of participants included in the SomaLogic assay at Visit 5 (n=5,327) and the exclusive availability of SomaLogic assay data from sample collected at Visit 3, SomaLogic data was used for the primary analysis. Of the 12 candidate proteins identified in this analysis, nine were also captured by the Olink platform (Supplemental Figure 6). For SomaLogic assay quality control, ARIC data sets were combined with data from healthy controls and samples were flagged if at least one of the four sample calibration factors was outside of the reference range of 0.4-2.5. These factors include hybridization control normalization factor, normalization factor for a dilution factor of 0.005%, normalization factor for a dilution factor of 0.5% and normalization factor for a dilution factor of 20%. Moreover, if at least one intraplate calibration factor was out of the reference range of 0.8-1.2, SOMAmers were flagged. DNA microarray technology was used to quantify the relative concentration levels of plasma proteins using 5,248 single-strand DNA-based aptamers that bind to targeted proteins based on a particular sequence or three-dimensional structure as previously published(*55*). Proteins levels were standardized to mean of 0 and standard deviation of 1. The ARIC study implemented additional quality control procedures. Detailed description of data standardization and quality control procedures have been previously published(*72*). To correct for skewness in the data distribution, SOMAmer measures were $\log_2$ transformed. Blind duplicates were run for 4% of participants in each visit, corresponding to 422 of 11565 individuals at visit 3 and 185 of 5327 individuals at visit 5. Bland-Altman analysis was employed to calculate the median inter-assay coefficient of variation (CV) for SOMAmers measured at each visit (Visit 3: 6.3%, Visit 5: 4.7). The median inter-assay coefficient of variation(*73, 55*) (CV) for SOMAmers measured at Visit 3 was 6.3%. Outliers were excluded for quality control and median split sample reliability coefficients were calculated for each visit (Visit 3: 0.85, Visit 5: 0.94). Six UniProt IDs and three protein names were completed by manual annotation. Aptamers were excluded from analysis if they had a CV > 50% (55 at Visit 3, 93 at Visit 5); had a variance of less than 0.01 on a log-scale (12 at visit 3, 12 at visit 5); or demonstrated binding to Fc mouse (228), contaminants (15), or non-proteins (70) including hybridization control elution, non-human proteins, non-biotin, non-cleavable, or spuriomer molecules. SOMAmer samples 5 times the standard deviation of the $\log_2$ scaled mean, were considered outliers and were winsorized.

Measurement of SES variables

The indicators used to create ADI included: education (proportion of people with <9 or ≥12 years of education, proportion of people who completed high school), median household income, income disparity, occupational composition (percentage of people in white collar occupations), percentage unemployed, percentage of families below poverty, percentage of single-parent households, percentage of home ownership, median home value, median rent, median mortgage, and household crowding, and factor score coefficients were used to weigh the indicators(*57, 58*). The ADI was created using factor analysis and principal component analysis from 17 socioeconomic indicators from US Census data. ADI is a validated measure of SES and has been used to assess individual health outcomes(*56*).

Assessment of covariates

Age, race, and gender were self-reported. ARIC participants undergo surveillance for coronary heart disease events including myocardial infarction or coronary intervention through review of hospital discharge codes, with subsequent chart abstraction and committee adjudication, as previously detailed(*74*). Hypertension was based on self-report of antihypertensive medication use, or a blood pressure measurement ≥140/90 mmHg. Diabetes mellitus was defined based on self-report of physician diagnosis, a fasting glucose >126 mg/dL, or a random glucose ≥200 mg/ dL. AF ascertainment was based on ECG at Visits 1 through 3, or hospital discharge records as previously described(*75*). eGFR was estimated by the 2021 CKD-EPI formula using serum creatinine and cystatin C concentrations(*76*) ($eGFR_{cr-cys}$) measured at Visit 3.

Exploratory factor analysis

Exploratory factor analysis was performed using the R package psych (v2.2.9). Education and income were modeled as ordinal categorical variables(*60*). Both variables were coded such that higher category reflected lower education and income, respectively. Neighborhood ADI was modeled as a continuous variable from 1 to 100, with higher ADI reflecting worse area deprivation. Spearman correlation was used to calculate their correlations. The factor analysis model was derived using the principal axis method and oblimin rotation. Regression factor scores were derived from the factor analysis model using the formula $XR^{-1}F$ where X is the data matrix (standardized to mean= 0 and standard deviation= 1), R is the correlation matrix, and F is the factor loading matrix. We used Kaiser-Meyer-Olkin (KMO) factor and Bartlett test for sampling adequacy. The KMO mean sample adequacy (MSA) and Bartlett's test for sphericity among Black (Income: 0.60, Education: 0.62, ADI: 0.73, p<0.0001) and white (Income: 0.61, Education: 0.63, ADI: 0.68, p<0.001) participants suggest that the data was appropriate for EFA.

Mediation Analysis

Mediation analysis was performed using the R package "mediation" (v4.5.0)(*61*). A linear regression model regressing protein levels on the SDOH factor adjusting for age, sex, BMI, eGFR, ever smoker, prevalent CAD, prevalent DM, prevalent AF, and prevalent hypertension was used for the mediator model. The outcome model was defined as an accelerated failure time model with Weibull survival distribution, regressing time to incident HF on SDOH factor, standardized protein levels, and their interaction while adjusting for the same set of covariates as in the mediator model. Standard errors in the outcome model were calculated by heteroscedasticity-consistent estimation of the covariance matrix. The interaction term was removed from the outcome model if p > 0.1. Ninety-five percent confidence intervals were calculated using quasi-Bayesian approximation with 1000 Monte Carlo draws. Proteins with significant average causal mediation effect (ACME) after Bonferroni correction based on number of proteins tested were subsequently identified among each race group.

Mendelian randomization

Analyses were run using the TwoSampleMR package in R(*77*). Summary statistics for HF were obtained from the HERMES consortium(*78*) and summary statistics for cardiac structure/function (left ventricular end diastolic volume [LVEDV], left ventricular end systolic volume [LVESV], left ventricular ejection fraction [LVEF]) were obtained from the UK Biobank(*79*). IV selection was based on genome-wide significance (p<5 x 10-8), and the chosen IVs were clumped with r^2 <0.001. For more than one IV, a casual estimate was calculated using inverse variance weighted (IVW) testing, while a Wald test was used for single IVs. Heterogeneity was tested using Cochran's heterogeneity test if more than two IVs were present. The MR Egger approach was used to check for the existence of horizontal pleiotropy if three or more IVs were present(*62*). The threshold for significant association was determined using Bonferroni correction for the number of independent sets of IVs tested: p<1.5e-3 for HERMES HF (0.05/33) and p<1.3e-3 for cardiac structure and function (0.05/38). We also performed a backward MR using IVs for HF as the exposure and IVs for proteins as the outcome to assess for potential reverse causality.

Colocalization analysis

We performed colocalization analysis, assuming a single causal variant, on significant MR hits located in a single genomic region using the R package coloc (v5.2.2)(*80, 81*). For each single region MR hit, the complete GWAS summary statistics were subset to within 500 kb of the SNP of interest. The default priors for the coloc.abf() function were used. Colocalization evidence was based on the posterior probability of H4 (Association with protein and outcome, through one shared SNP) from software output, with sufficient evidence of colocalization denoted by a threshold of 75%. If hypothesis H3 (Association with protein and outcome, but with two independent SNPs) reached a threshold of 75% or greater, it was

concluded that the protein and phenotype of interest were likely driven by different causal variants, not supporting colocalization.

Tissue expression analysis

Genotype-tissue expression data from the Genotype-Tissue Expression (GTEx; project [V8]; https://gtexportal.org/home/datasets) in healthy tissue were used to assess tissue-specific gene expression for genes coding for candidate proteins(*82*). Hierarchical clustering was used to identify groups of proteins based on gene expression across tissues.

UK Biobank Replication Analysis

The SDOH included in the UK Biobank replication analysis included: household income, educational attainment, and Townsend Deprivation Index (TDI). Household income was defined as the total household income before taxes and was self-reported by participants into the following categories: £18,000 or less, £18,000 – 30,000, £31,000 – 51,999, £52,000 – 100,000, greater than £100,000, don't know, or prefer not to answer(67). Educational attainment was self-reported by participants in the following categories: College or university degree, NVQ/HND/HNC or equivalent, other professional training (such as nursing or teaching), A levels, GCSEs, CSEs, no qualifications, or prefer not to answer(68). The TDI is a measure of area deprivation based on participant postal code, and is comprised of factors such as unemployment, non-car ownership, non-home ownership, and household overcrowding as previously described(69-71). UK Biobank application number #285361.

Fig. S1. Distribution of income, education, and ADI by race category at Visit 3.

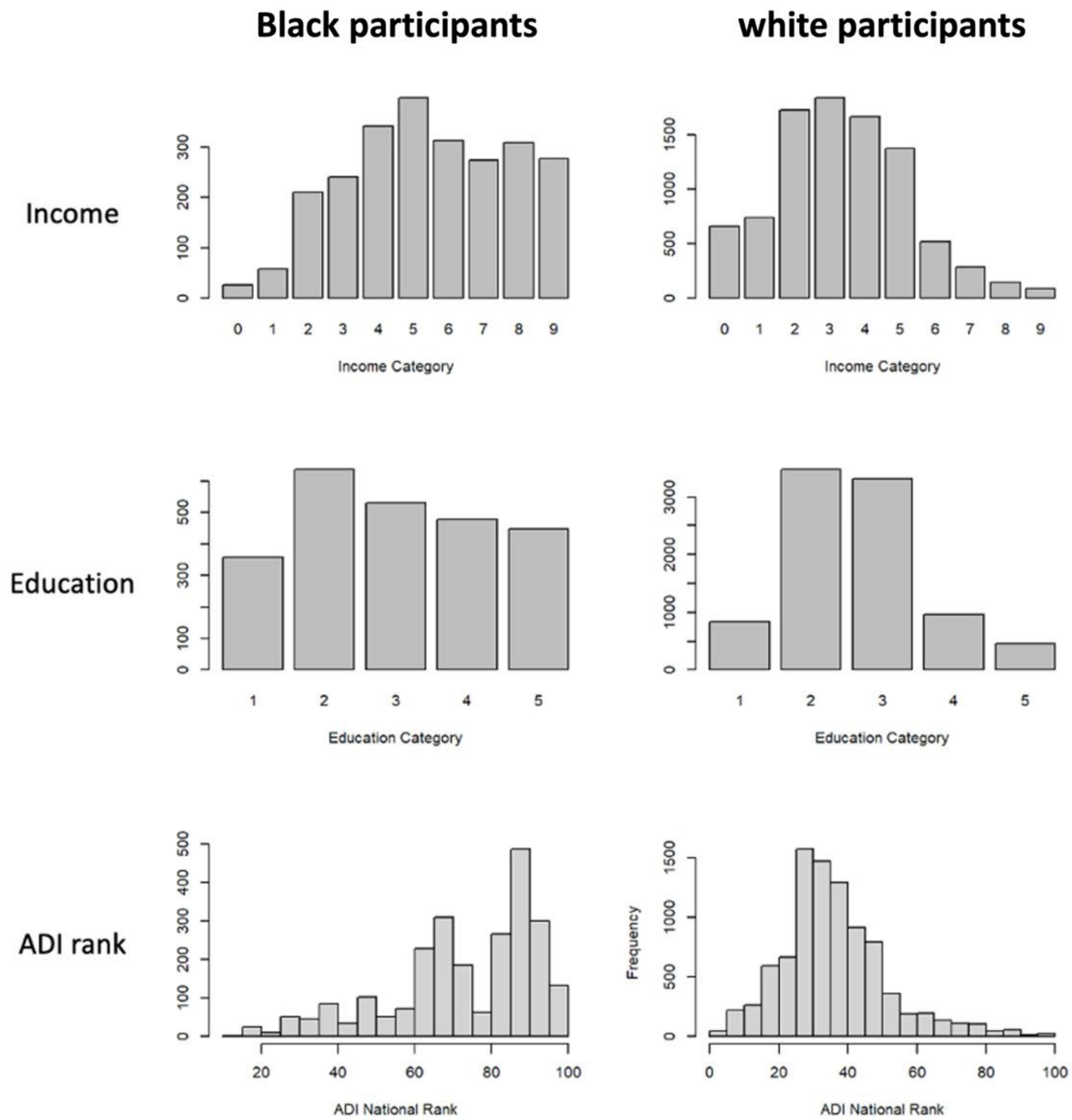

Supplemental Results

Fig. S2. Sensitivity analysis adjusting for field center in associations between proteins with HF.

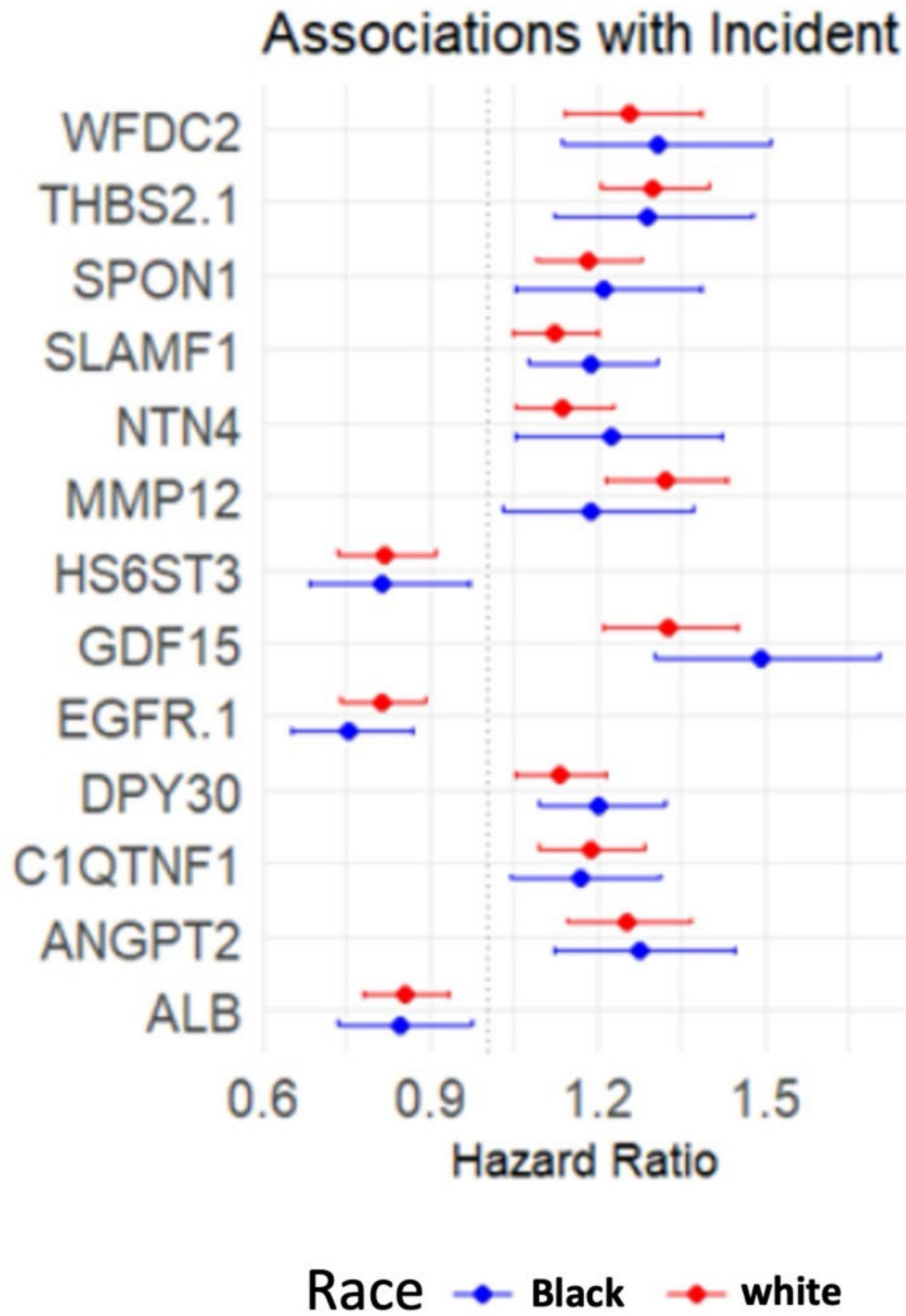

In a sensitivity analysis further adjusting for ARIC field center in addition to demographics and comorbidities, 38 of 80 proteins in the Black race stratum and 131 of 881 proteins in the white race stratum demonstrated FDR significance in associations with HF. Of these, 13 proteins were shared across race groups, including the 12 candidate proteins reported in the primary analysis and MMP12 as an additional shared protein.

Fig. S3. Tissue expression of the 12 SDOH and incident HF-associated proteins

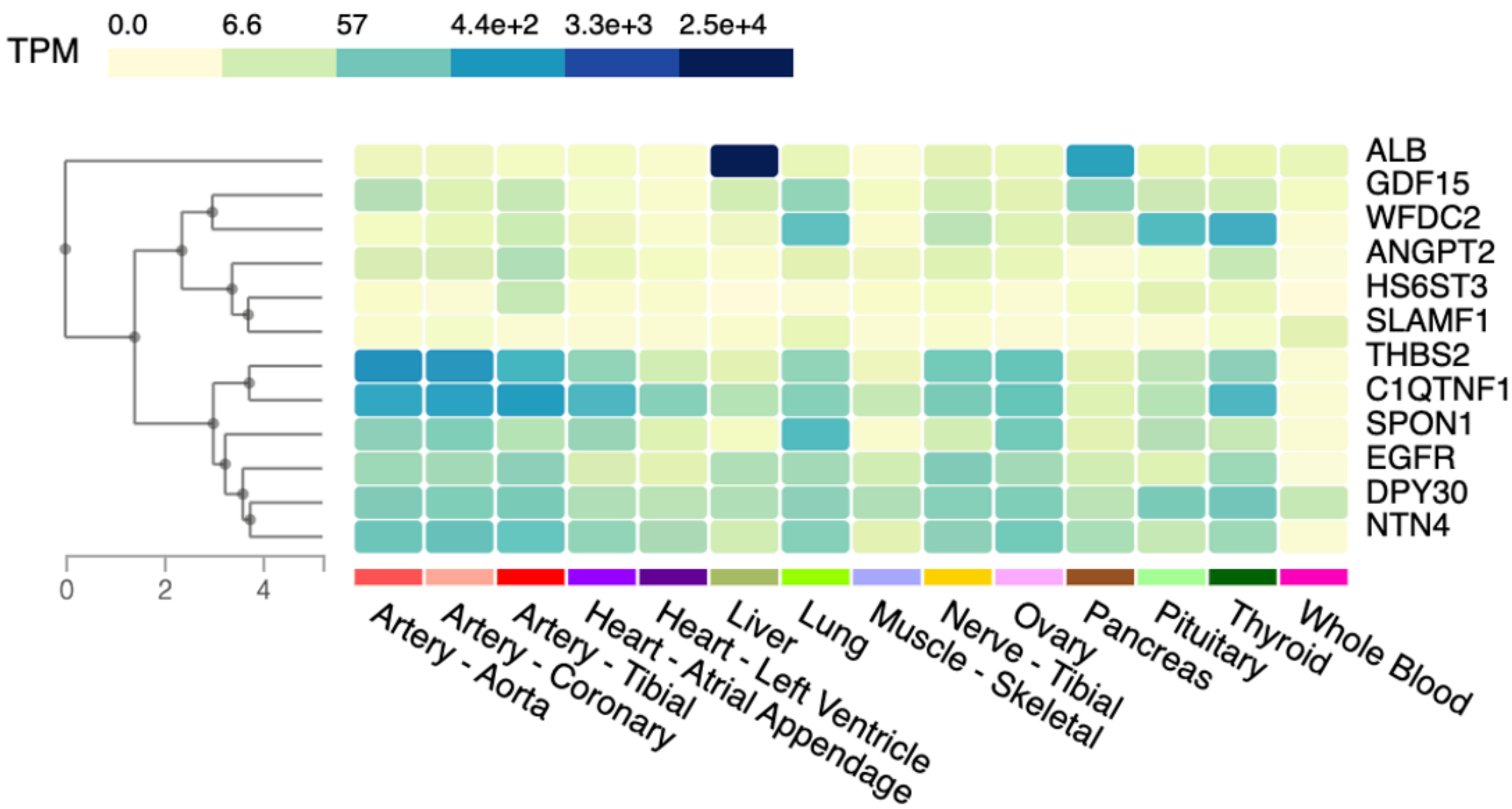

Fig. S4. Mendelian Randomization. (A) Mendelian randomization analysis results of proteogenomic analyses to assess for potential causal associations of proteins with myocardial fibrosis (MIF). **(B)** Summary of results of MR for MIF including beta coefficient, standard error, p-value, and number of SNPs

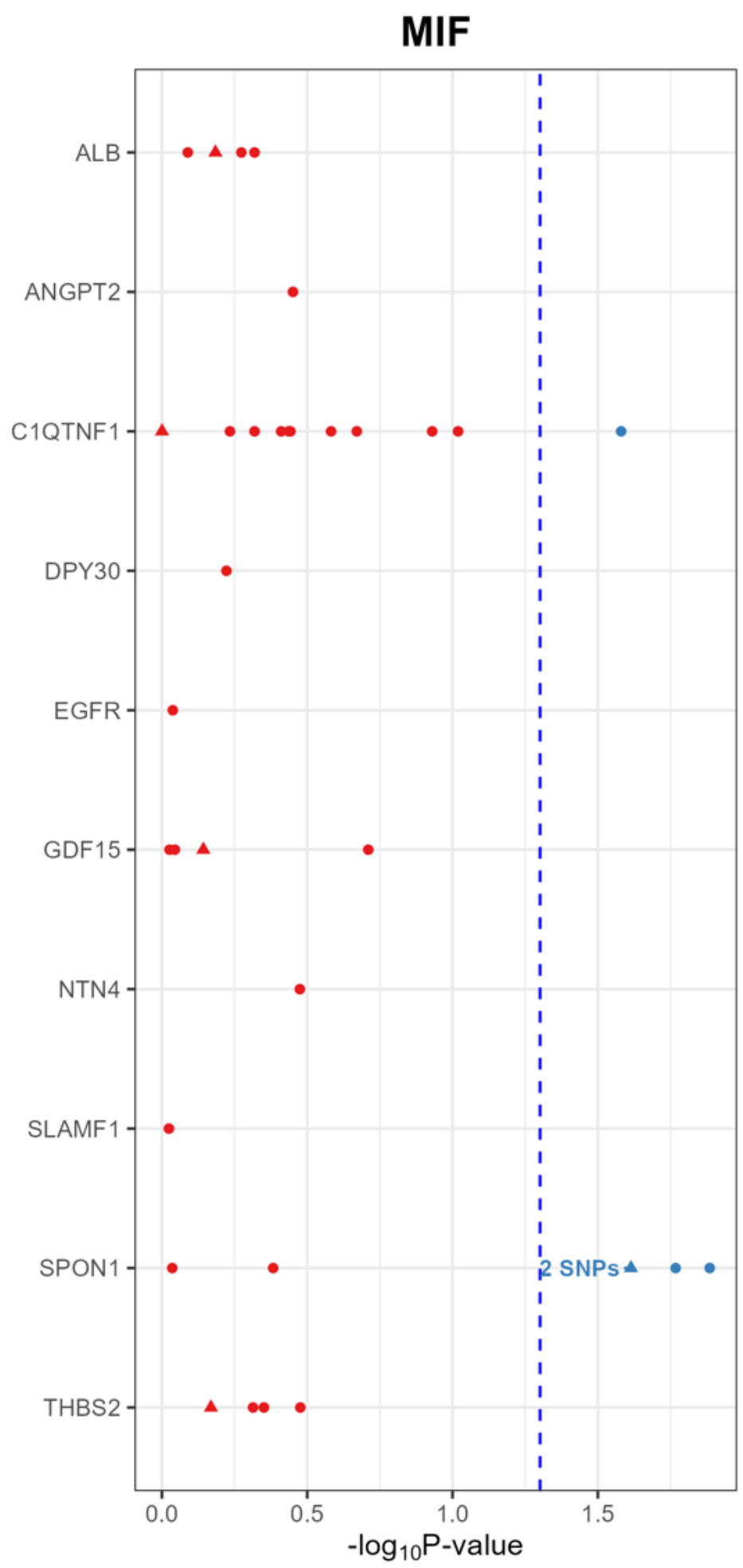

Gene	Outcome	Method	Beta	SE	P value	# of SNPs	SNP	Source	Term	name	Type
SPON1	MIF	Wald ratio	0.04196105	0.01759341	0.01707711	1	rs10832169	Database	SeqId_4297_62	Spondin-1	Single SNP (cis)
SPON1	MIF	Wald ratio	0.04532263	0.0182542	0.0130332	1	rs1969539	Science Paper	SeqId_4297_62	Spondin-1	Single SNP (cis)
C1QTNF1	MIF	Wald ratio	-0.0662408	0.02981414	0.0262973	1	rs5848	Database	SeqId_6304_8	Complement C1q tumor necrosis factor-related protein 1	Single SNP (trans)
SPON1	MIF	Inverse variance weighted	0.03680158	0.01634212	0.02432565	2	rs112635299; rs138185133; rs45543339; rs17288108; rs10832169; rs117864595; rs116503616; rs73043857; rs10762481; rs112635299; rs5848; rs646776; rs72802395	Database	SeqId_4297_62	Spondin-1	Multiple SNPs (2 SNPs)

Fig. S5. Correlation of UK Biobank SDOH measures and derivation and prognostic relevance of SDOH factor at the baseline assessment. Panel **(A)** Spearman correlation matrix among SDOH variables; Panel **(B)** Principal axis exploratory factor loadings; Panel **(C)** Association of factor score with risk of incident HF in Cox proportional hazard models adjusted for age, sex, center, BMI, eGFR, smoking status, CAD, AF, DM and HTN

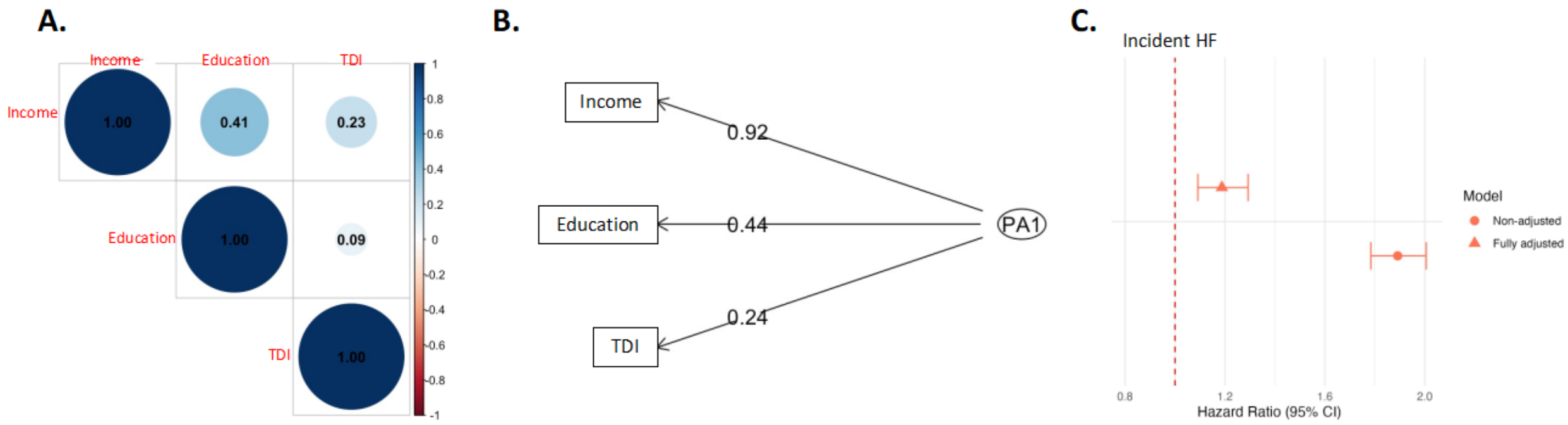

Fig. S6. Cross-platform concordance of proteomic metrics captured with the Somalogic (ARIC) and Olink (UK Biobank) platforms

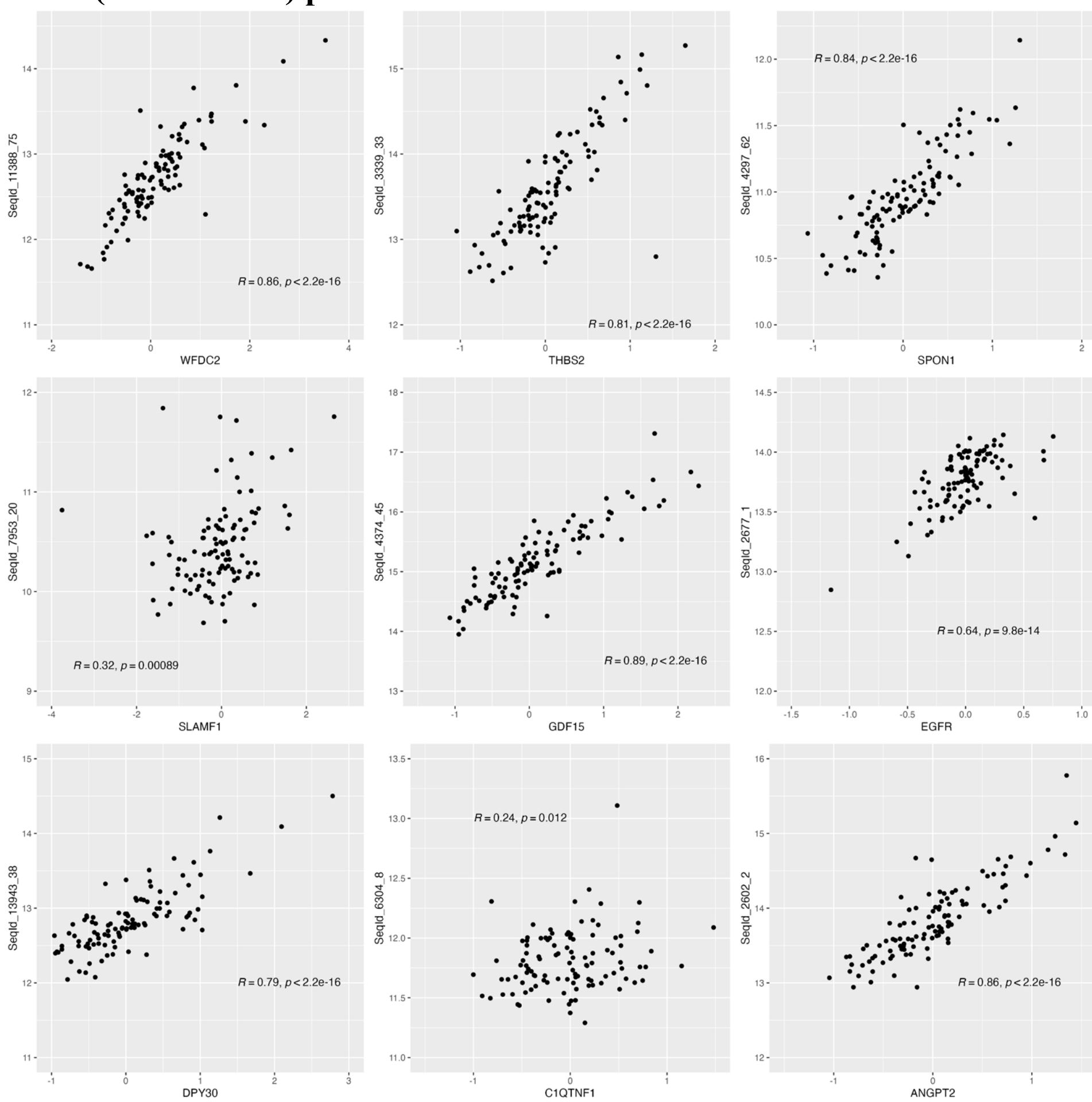

REFERENCES

1. F. B. Ahmad, J. A. Cisewski, A. Miniño, R. N. Anderson, Provisional mortality data—United States, 2020. *MMWR Morb. Mortal Wkly Rep.* **70**, 519–522 (2021).

2. A. M. Clark, M. DesMeules, W. Luo, A. S. Duncan, A. Wielgosz, Socioeconomic status and cardiovascular disease: Risks and implications for care. *Nat. Rev. Cardiol.* **6**, 712–722 (2009).

3. E. Ruel, E. N. Reither, S. A. Robert, P. M. Lantz, Neighborhood effects on BMI trends: Examining BMI trajectories for Black and White women. *Health Place* **16**, 191–198 (2010).

4. D. R. Buys, V. J. Howard, L. A. McClure, K. C. Buys, P. Sawyer, R. M. Allman, E. B. Levitan, Association between neighborhood disadvantage and hypertension prevalence, awareness, treatment, and control in older adults: Results from the University of Alabama at Birmingham study of aging. *Am. J. Public Health* **105**, 1181–1188 (2015).

5. L. Sheets, G. F. Petroski, J. Jaddoo, Y. Barnett, C. Barnett, L. E. Kelley, V. Raman, A. J. Kind, The effect of neighborhood disadvantage on diabetes prevalence. *AMIA Annu. Symp. Proc.* **2017**, 1547–1553 (2018).

6. J. I. Halonen, S. Stenholm, J. Pentti, I. Kawachi, S. V. Subramanian, M. Kivimäki, J. Vahtera, Childhood psychosocial adversity and adult neighborhood disadvantage as predictors of cardiovascular disease: A cohort study. *Circulation* **132**, 371–379 (2015).

7. S. Barber, D. A. Hickson, X. Wang, M. Sims, C. Nelson, A. V. Diez-Roux, Neighborhood disadvantage, poor social conditions, and cardiovascular disease incidence among african american adults in the jackson heart study. *Am. J. Public Health* **106**, 2219–2226 (2016).

8. N. M. Hawkins, P. S. Jhund, J. J. V. McMurray, S. Capewell, Heart failure and socioeconomic status: Accumulating evidence of inequality. *Eur. J. Heart Fail.* **14**, 138–146 (2012).

9. Y. C. Cozier, M. A. Albert, N. Castro-Webb, P. F. Coogan, P. Ridker, H. W. Kaufman, J. R. Palmer, L. Rosenberg, Neighborhood socioeconomic status in relation to serum biomarkers in the black women's health study. *J. Urban Health* **93**, 279–291 (2016).

10. K. A. Muscatell, S. N. Brosso, K. L. Humphreys, Socioeconomic status and inflammation: A meta-analysis. *Mol. Psychiatry* **25**, 2189–2199 (2020).

11. T. Seeman, S. S. Merkin, E. Crimmins, B. Koretz, S. Charette, A. Karlamangla, Education, income and ethnic differences in cumulative biological risk profiles in a national sample of US adults: NHANES III (1988–1994). *Soc. Sci. Med.* **66**, 72–87 (2008).

12. S. Waheed, K. Matsushita, Y. Sang, R. Hoogeveen, C. Ballantyne, J. Coresh, B. C. Astor, Combined association of albuminuria and cystatin C–based estimated GFR with mortality, coronary heart disease, and heart failure outcomes: The atherosclerosis risk in communities (ARIC) study. *Am. J. Kidney Dis.* **60**, 207–216 (2012).

13. P. Vart, R. T. Gansevoort, M. M. Joosten, U. Bültmann, S. A. Reijneveld, Socioeconomic disparities in chronic kidney disease. *Am. J. Prev. Med.* **48**, 580–592 (2015).

14. S. Cohen, W. J. Doyle, A. Baum, Socioeconomic status is associated with stress hormones. *Psychosom. Med.* **68**, 414–420 (2006).

15. B. McEwen, Stress and the individual: Mechanisms leading to disease. *Arch. Intern. Med.* **153**, 2093–2101 (1993).

16. B. S. McEwen, Protective and damaging effects of stress mediators. *N. Engl. J. Med.* **338**, 171–179 (1998).

17. G. C. Gee, C. L. Ford, Structural racism and health inequities: Old issues, new directions. *Du Bois Rev.* **8**, 115–132 (2011).

18. B. K. Obach, Demonstrating the social construction of race. *Teach. Sociol.* **27**, 252 (1999).

19. S. N. Bleich, M. G. Findling, L. S. Casey, R. J. Blendon, J. M. Benson, G. K. SteelFisher, J. M. Sayde, C. Miller, Discrimination in the United States: Experiences of black Americans. *Health Serv. Res.* **54**, 1399–1408 (2019).

20. C. Wildeman, E. A. Wang, Mass incarceration, public health, and widening inequality in the USA. *Lancet* **389**, 1464–1474 (2017).

21. T. Hobson-Prater, T. G. J. Leech, The significance of race for neighborhood social cohesion: Perceived difficulty of collective action in majority black neighborhoods. *J. Soc. Soc. Welfare* **39**, 89–109 (2012).

22. Z. Hajnal, N. Lajevardi, L. Nielson, Voter identification laws and the suppression of minority votes. *J. Polit.* **79**, 363–379 (2017).

23. M. M. Safford, E. Reshetnyak, M. R. Sterling, J. S. Richman, P. M. Muntner, R. W. Durant, J. Booth, L. C. Pinheiro, Number of social determinants of health and fatal and nonfatal incident coronary heart disease in the REGARDS study. *Circulation* **143**, 244–253 (2021).

24. L. C. Pinheiro, E. Reshetnyak, M. R. Sterling, E. B. Levitan, M. M. Safford, P. Goyal, Multiple vulnerabilities to health disparities and incident heart failure hospitalization in the REGARDS study. *Circ. Cardiovasc. Qual. Outcomes* **13**, e006438 (2020).

25. G. Bey, J. Pike, P. Palta, A. Zannas, Q. Xiao, S.-A. Love, G. Heiss, Biological age mediates the effects of perceived neighborhood problems on heart failure risk among black persons. *J. Racial Ethn. Health Disparities* **10**, 3018–3030 (2023).

26. A. Mentias, M. S. Mujahid, A. Sumarsono, R. K. Nelson, J. M. Madron, T. M. Powell-Wiley, U. R. Essien, N. Keshvani, S. Girotra, A. A. Morris, M. Sims, Q. t. Capers, C. Yancy, M. Y. Desai, V. Menon, S. Rao, A. Pandey, Historical redlining, socioeconomic distress, and risk of heart failure among medicare beneficiaries. *Circulation* **148**, 210–219 (2023).

27. M. Pietzner, E. Wheeler, J. Carrasco-Zanini, A. Cortes, M. Koprulu, M. A. Wörheide, E. Oerton, J. Cook, I. D. Stewart, N. D. Kerrison, J. a. Luan, J. Raffler, M. Arnold, W. Arlt, S. O'Rahilly, G. Kastenmüller, E. R. Gamazon, A. D. Hingorani, R. A. Scott, N. J. Wareham, C. Langenberg, Mapping the proteo-genomic convergence of human diseases. *Science* **374**, eabj1541 (2021).

28. M. Ramanjaneya, J. Jerobin, I. Bettahi, K. S. Siveen, A.-B. Abou-Samra, Emerging roles of C1Q tumor necrosis factor-related proteins in metabolic diseases. *Trans. Med. Commun.* **6**, 5 (2021).

29. G. Bai, X. Xie, H. Liu, D. Qiang, Y. He, L. Zhang, X. Zhang, The association between complement C1q tumour necrosis factor-related protein-1 (CTRP-1) level and metabolic syndrome. *Endokrynol. Pol.* **74**, 271–276 (2023).

30. R. Song, W. Hu, R. Cheng, Y. Zhao, W. Qin, X. Li, Y. Zhu, L. Gan, J. Liu, Association between circulating levels of C1q/TNF-related protein-9 and type 2 diabetes mellitus: A systematic review and meta-analysis. *J. Clin. Endocrinol. Metab.* **108**, 2728–2738 (2023).

31. C. Li, J. W. Chen, Z. H. Liu, Y. Shen, F. H. Ding, G. Gu, J. Liu, J. P. Qiu, J. Gao, R. Y. Zhang, W. F. Shen, X. Q. Wang, L. Lu, CTRP5 promotes transcytosis and oxidative modification of low-density lipoprotein and the development of atherosclerosis. *Atherosclerosis* **278**, 197–209 (2018).

32. Z.-Z. Zhang, G. Wang, S.-H. Yin, X.-H. Yu, C1q tumor necrosis factor–related protein 1: A promising therapeutic target for atherosclerosis. *J. Cardiovasc. Pharmacol.* **79**, 273–280 (2022).

33. P. Shabani, H. Naeimi Khaledi, M. Beigy, S. Emamgholipour, E. Parvaz, H. Poustchi, M. Doosti, Circulating level of CTRP1 in patients with nonalcoholic fatty liver disease (NAFLD): Is it through insulin resistance? *PLOS ONE* **10**, e0118650 (2015).

34. Y. Yang, S. Liu, R.-Y. Zhang, H. Luo, L. Chen, W.-F. He, R. Lei, M.-R. Liu, H.-X. Hu, M. Chen, Association between C1q/TNF-related protein-1 levels in human plasma and epicardial adipose tissues and congestive heart failure. *Cell Physiol. Biochem.* **42**, 2130–2143 (2017).

35. H. Hu, C. Li, S. Ying, X. Wu, T. Zhu, Q. Zhou, Y. Zhang, Y. Liu, R. Zhu, CTRP1 aggravates cardiac fibrosis via regulating the NOX2/P38 pathway in macrophage. *Cell J.* **24**, 732–740 (2022).

36. R. Kvetnansky, X. Lu, M. G. Ziegler, “Stress-triggered changes in peripheral catecholaminergic systems” in *Advances in Pharmacology* (Elsevier, 2013), vol. 68, pp. 359–397.

37. X. Q. Wang, Z. H. Liu, L. Xue, L. Lu, J. Gao, Y. Shen, K. Yang, Q. J. Chen, R. Y. Zhang, W. F. Shen, C1q/TNF-related protein 1 links macrophage lipid metabolism to inflammation and atherosclerosis. *Atherosclerosis* **250**, 38–45 (2016).

38. S. Heximer, M. Husain, A candidate hypertension gene. *Circ. Res.* **100**, 940–942 (2007).

39. J.-R. Clemitson, R. J. Dixon, S. Haines, A. J. Bingham, B. R. Patel, L. Hall, M. Lo, J. Sassard, F. J. Charchar, N. J. Samani, Genetic dissection of a blood pressure quantitative trait locus on rat chromosome 1 and gene expression analysis identifies SPON1 as a novel candidate hypertension gene. *Circ. Res.* **100**, 992–999 (2007).

40. J. Barallobre-Barreiro, A. Didangelos, F. A. Schoendube, I. Drozdov, X. Yin, M. Fernández-Caggiano, P. Willeit, V. O. Puntmann, G. Aldama-López, A. M. Shah, N. Doménech, M. Mayr, Proteomics analysis of cardiac extracellular matrix remodeling in a porcine model of ischemia/reperfusion injury. *Circulation* **125**, 789–802 (2012).

41. T. J. Wang, K. C. Wollert, M. G. Larson, E. Coglianese, E. L. McCabe, S. Cheng, J. E. Ho, M. G. Fradley, A. Ghorbani, V. Xanthakis, T. Kempf, E. J. Benjamin, D. Levy, R. S. Vasan, J. L. Januzzi, Prognostic utility of novel biomarkers of cardiovascular stress: The framingham heart study. *Circulation* **126**, 1596–1604 (2012).

42. T. Kempf, S. Von Haehling, T. Peter, T. Allhoff, M. Cicoira, W. Doehner, P. Ponikowski, G. S. Filippatos, P. Rozentryt, H. Drexler, S. D. Anker, K. C. Wollert, Prognostic utility of growth differentiation factor-15 in patients with chronic heart failure. *J. Am. Coll. Cardiol.* **50**, 1054–1060 (2007).

43. M. M. Y. Chan, R. Santhanakrishnan, J. P. C. Chong, Z. Chen, B. C. Tai, O. W. Liew, T. P. Ng, L. H. Ling, D. Sim, K. T. G. Leong, P. S. D. Yeo, H.-Y. Ong, F. Jaufeerally, R. C.-C. Wong, P. Chai, A. F. Low, A. M. Richards, C. S. P. Lam, Growth differentiation factor 15 in heart failure with preserved vs. reduced ejection fraction. *Eur. J. Heart Fail.* **18**, 81–88 (2016).

44. V. Xanthakis, M. G. Larson, K. C. Wollert, J. Aragam, S. Cheng, J. Ho, E. Coglianese, D. Levy, W. S. Colucci, G. Michael Felker, E. J. Benjamin, J. L. Januzzi, T. J. Wang, R. S.

Vasan, Association of novel biomarkers of cardiovascular stress with left ventricular hypertrophy and dysfunction: Implications for screening. *J. Am. Heart Assoc.* **2**, e000399 (2013).

45. I. Hellström, J. Raycraft, M. Hayden-Ledbetter, J. A. Ledbetter, M. Schummer, M. McIntosh, C. Drescher, N. Urban, K. E. Hellström, The HE4 (WFDC2) protein is a biomarker for ovarian carcinoma. *Cancer Res.* **63**, 3695–3700 (2003).

46. A. Piek, W. C. Meijers, N. F. Schroten, R. T. Gansevoort, R. A. De Boer, H. H. W. Silljé, HE4 serum levels are associated with heart failure severity in patients with chronic heart failure. *J. Card. Fail.* **23**, 12–19 (2017).

47. L. Lind, D. Zanetti, M. Ingelsson, S. Gustafsson, J. Ärnlöv, T. L. Assimes, Large-scale plasma protein profiling of incident myocardial infarction, ischemic stroke, and heart failure. *J. Am. Heart Assoc.* **10**, e023330 (2021).

48. C. Kirchhoff, I. Habben, R. Ivell, N. Krull, A major human epididymis-specific cDNA encodes a protein with sequence homology to extracellular proteinase inhibitors. *Biol. Reprod.* **45**, 350–357 (1991).

49. J. Punnonen, B. G. Cocks, J. M. Carballido, B. Bennett, D. Peterson, G. Aversa, J. E. D. Vries, Soluble and membrane-bound forms of signaling lymphocytic activation molecule (SLAM) induce proliferation and Ig synthesis by activated human B lymphocytes. *J. Exp. Med.* **185**, 993–1004 (1997).

50. B. S. Peplinski, B. A. Houston, D. A. Bluemke, S. M. Kawut, T. M. Kolb, R. A. Kronmal, J. A. C. Lima, D. D. Ralph, S. G. Rayner, Z. L. Steinberg, R. J. Tedford, P. J. Leary, Associations of angiopoietins with heart failure incidence and severity. *J. Card. Fail.* **27**, 786–795 (2021).

51. A. J. Piper-Vallillo, D. B. Costa, M. A. Sabe, A. Asnani, Heart failure associated with the epidermal growth factor receptor inhibitor osimertinib. *JACC CardioOncology* **2**, 119–122 (2020).

52. K. Lemmens, K. Doggen, G. W. De Keulenaer, Role of neuregulin-1/ErbB signaling in cardiovascular physiology and disease: Implications for therapy of heart failure. *Circulation* **116**, 954–960 (2007).

53. D. H. Katz, U. A. Tahir, D. Ngo, M. D. Benson, Y. Gao, X. Shi, M. Nayor, M. J. Keyes, M. G. Larson, M. E. Hall, A. Correa, S. Sinha, D. Shen, M. Herzig, Q. Yang, J. M. Robbins, Z.-Z. Chen, D. E. Cruz, B. Peterson, R. S. Vasan, T. J. Wang, J. G. Wilson, R. E. Gerszten, Multiomic profiling in black and white populations reveals novel candidate pathways in left ventricular hypertrophy and incident heart failure specific to black adults. *Circ. Genom. Precis. Med.* **14**, e003191 (2021).

54. J. D. Wright, A. R. Folsom, J. Coresh, A. R. Sharrett, D. Couper, L. E. Wagenknecht, T. H. Mosley, C. M. Ballantyne, E. A. Boerwinkle, W. D. Rosamond, G. Heiss, The ARIC (atherosclerosis risk in communities) study: JACC focus seminar 3/8. *J. Am. Coll. Cardiol.* **77**, 2939–2959 (2021).

55. K. A. Walker, J. Chen, J. Zhang, M. Fornage, Y. Yang, L. Zhou, M. E. Grams, A. Tin, N. Daya, R. C. Hoogeveen, A. Wu, K. J. Sullivan, P. Ganz, S. L. Zeger, E. F. Gudmundsson, V. Emilsson, L. J. Launer, L. L. Jennings, V. Gudnason, N. Chatterjee, R. F. Gottesman, T. H. Mosley, E. Boerwinkle, C. M. Ballantyne, J. Coresh, Large-scale plasma proteomic analysis identifies proteins and pathways associated with dementia risk. *Nat. Aging* **1**, 473–489 (2021).

56. A. V. Diez-Roux, F. J. Nieto, H. A. Tyroler, L. D. Crum, M. Szklo, Social inequalities and atherosclerosis the atherosclerosis risk in communities study. *Am. J. Epidemiol.* **141**, 960–972 (1995).

57. G. K. Singh, Area deprivation and widening inequalities in US mortality, 1969–1998. *Am. J. Public Health* **93**, 1137–1143 (2003).

58. A. J. H. Kind, W. R. Buckingham, Making neighborhood-disadvantage metrics accessible—The neighborhood atlas. *N. Engl. J. Med.* **378**, 2456–2458 (2018).

59. L. R. Loehr, W. D. Rosamond, P. P. Chang, A. R. Folsom, L. E. Chambless, Heart failure incidence and survival (from the atherosclerosis risk in communities study). *Am. J. Cardiol.* **101**, 1016–1022 (2008).

60. R. R. William, psych: Procedures for Personality and Psychological Research—Northwestern Scholars (2017).

61. D. Tingley, T. Yamamoto, K. Hirose, L. Keele, K. Imai, mediation: R package for causal mediation analysis. *J. Stat. Soft.* **59**, 1–38 (2014).

62. J. Bowden, G. D. Smith, S. Burgess, Mendelian randomization with invalid instruments: Effect estimation and bias detection through Egger regression. *Int. J. Epidemiol.* **44**, 512–525 (2015).

63. B. B. Sun, J. C. Maranville, J. E. Peters, D. Stacey, J. R. Staley, J. Blackshaw, S. Burgess, T. Jiang, E. Paige, P. Surendran, C. Oliver-Williams, M. A. Kamat, B. P. Prins, S. K. Wilcox, E. S. Zimmerman, A. Chi, N. Bansal, S. L. Spain, A. M. Wood, N. W. Morrell, J. R. Bradley, N. Janjic, D. J. Roberts, W. H. Ouwehand, J. A. Todd, N. Soranzo, K. Suhre, D. S. Paul, C. S. Fox, R. M. Plenge, J. Danesh, H. Runz, A. S. Butterworth, Genomic atlas of the human plasma proteome. *Nature* **558**, 73–79 (2018).

64. A. Gudjonsson, V. Gudmundsdottir, G. T. Axelsson, E. F. Gudmundsson, B. G. Jonsson, L. J. Launer, J. R. Lamb, L. L. Jennings, T. Aspelund, V. Emilsson, V. Gudnason, A genome-wide association study of serum proteins reveals shared loci with common diseases. *Nat. Commun.* **13**, 480 (2022).

65. C. Sudlow, J. Gallacher, N. Allen, V. Beral, P. Burton, J. Danesh, P. Downey, P. Elliott, J. Green, M. Landray, B. Liu, P. Matthews, G. Ong, J. Pell, A. Silman, A. Young, T. Sprosen, T. Peakman, R. Collins, UK Biobank: An open access resource for identifying the causes of a wide range of complex diseases of middle and old age. *PLOS Med.* **12**, e1001779 (2015).

66. H. Taylor, M. Lewins, M. G. B. Foody, O. Gray, J. Besevic, M. C. Conroy, R. Collins, B. Lacey, N. Allen, L. Burkitt-Gray, UK Biobank—A unique resource for discovery and translation research on genetics and neurologic disease. *Neurol. Genet.* **11**, e200226 (2025).

67. biobank, Data-Field 738. https://biobank.ctsu.ox.ac.uk/crystal/field.cgi?id=738.

68. biobank, Data-Field 6138. https://biobank.ctsu.ox.ac.uk/crystal/field.cgi?id=6138.

69. biobank, Data-Field 22189. https://biobank.ctsu.ox.ac.uk/crystal/field.cgi?id=22189.

70. P. Townsend, Deprivation. *J. Soc. Policy* **16**, 125–146 (1987).

71. P. Townsend, P. Phillimore, A. Beattie, *Health and Deprivation: Inequality and the North* (Routledge, 2023).

72. L. Gold, D. Ayers, J. Bertino, C. Bock, A. Bock, E. N. Brody, J. Carter, A. B. Dalby, B. E. Eaton, T. Fitzwater, D. Flather, A. Forbes, T. Foreman, C. Fowler, B. Gawande, M. Goss, M. Gunn, S. Gupta, D. Halladay, J. Heil, J. Heilig, B. Hicke, G. Husar, N. Janjic, T. Jarvis, S. Jennings, E. Katilius, T. R. Keeney, N. Kim, T. H. Koch, S. Kraemer, L. Kroiss, N. Le, D. Levine, W. Lindsey, B. Lollo, W. Mayfield, M. Mehan, R. Mehler, S. K. Nelson, M. Nelson, D. Nieuwlandt, M. Nikrad, U. Ochsner, R. M. Ostroff, M. Otis, T. Parker, S. Pietrasiewicz, D. I. Resnicow, J. Rohloff, G. Sanders, S. Sattin, D. Schneider, B. Singer, M. Stanton, A. Sterkel, A. Stewart, S. Stratford, J. D. Vaught, M. Vrkljan, J. J. Walker, M. Watrobka, S. Waugh, A. Weiss, S. K. Wilcox, A. Wolfson, S. K. Wolk, C. Zhang, D. Zichi, Aptamer-based multiplexed proteomic technology for biomarker discovery. *PLOS ONE* **5**, e15004 (2010).

73. A. M. Shah, P. L. Myhre, V. Arthur, P. Dorbala, H. Rasheed, L. F. Buckley, B. Claggett, G. Liu, J. Ma, N. Q. Nguyen, K. Matsushita, C. Ndumele, A. Tin, K. Hveem, C. Jonasson, H. Dalen, E. Boerwinkle, R. C. Hoogeveen, C. Ballantyne, J. Coresh, T. Omland, B. Yu, Large scale plasma proteomics identifies novel proteins and protein networks associated with heart failure development. *Nat. Commun.* **15**, 528 (2024).

74. A. D. White, A. R. Folsom, L. E. Chambless, A. R. Sharret, K. Yang, D. Conwill, M. Higgins, O. D. Williams, H. A. Tyroler, A. I. The, Community surveillance of coronary heart disease in the atherosclerosis risk in communities (ARIC) study: Methods and initial two years' experience. *J. Clin. Epidemiol.* **49**, 223–233 (1996).

75. A. Alonso, S. K. Agarwal, E. Z. Soliman, M. Ambrose, A. M. Chamberlain, R. J. Prineas, A. R. Folsom, Incidence of atrial fibrillation in whites and African-Americans: The atherosclerosis risk in communities (ARIC) study. *Am. Heart J.* **158**, 111–117 (2009).

76. L. A. Inker, C. H. Schmid, H. Tighiouart, J. H. Eckfeldt, H. I. Feldman, T. Greene, J. W. Kusek, J. Manzi, F. Van Lente, Y. L. Zhang, J. Coresh, A. S. Levey, C.-E. Investigators, Estimating glomerular filtration rate from serum creatinine and cystatin C. *N. Engl. J. Med.* **367**, 20–29 (2012).

77. G. Hemani, J. Zheng, B. Elsworth, K. H. Wade, V. Haberland, D. Baird, C. Laurin, S. Burgess, J. Bowden, R. Langdon, V. Y. Tan, J. Yarmolinsky, H. A. Shihab, N. J. Timpson, D. M. Evans, C. Relton, R. M. Martin, G. Davey Smith, T. R. Gaunt, P. C. Haycock, The MR-Base platform supports systematic causal inference across the human phenome. *eLife* **7**, e34408 (2018).

78. S. Shah, A. Henry, C. Roselli, H. Lin, G. Sveinbjornsson, G. Fatemifar, A. K. Hedman, J. B. Wilk, M. P. Morley, M. D. Chaffin, A. Helgadottir, N. Verweij, A. Dehghan, P. Almgren, C. Andersson, K. G. Aragam, J. Arnlov, J. D. Backman, M. L. Biggs, H. L. Bloom, J. Brandimarto, M. R. Brown, L. Buckbinder, D. J. Carey, D. I. Chasman, X. Chen, X. Chen, J. Chung, W. Chutkow, J. P. Cook, G. E. Delgado, S. Denaxas, A. S. Doney, M. Dörr, S. C. Dudley, M. E. Dunn, G. Engström, T. Esko, S. B. Felix, C. Finan, I. Ford, M. Ghanbari, S. Ghasemi, V. Giedraitis, F. Giulianini, J. S. Gottdiener, S. Gross, D. F. Guðbjartsson, R. Gutmann, C. M. Haggerty, P. van der Harst, C. L. Hyde, E. Ingelsson, J. W. Jukema, M. Kavousi, K.-T. Khaw, M. E. Kleber, L. Køber, A. Koekemoer, C. Langenberg, L. Lind, C. M. Lindgren, B. London, L. A. Lotta, R. C. Lovering, J. Luan, P. Magnusson, A. Mahajan, K. B. Margulies, W. März, O. Melander, I. R. Mordi, T. Morgan, A. D. Morris, A. P. Morris, A. C. Morrison, M. W. Nagle, C. P. Nelson, A. Niessner, T. Niiranen, M. L. O'Donoghue, A. T. Owens, C. N. A. Palmer, H. M. Parry, M. Perola, E. Portilla-Fernandez, B. M. Psaty, R. G. Center, K. M. Rice, P. M. Ridker, S. P. R. Romaine, J. I. Rotter, P. Salo, V. Salomaa, J. van Setten, A. A. Shalaby, D. T. Smelser, N. L. Smith, S. Stender, D. J. Stott, P. Svensson, M.-L. Tammesoo, K. D. Taylor, M. Teder-Laving, A. Teumer, G. Thorgeirsson, U. Thorsteinsdottir, C. Torp-Pedersen, S. Trompet, B. Tyl, A. G. Uitterlinden, A. Veluchamy, U. Völker, A. A. Voors, X. Wang, N. J. Wareham, D. Waterworth, P. E. Weeke, R. Weiss, K. L. Wiggins, H.

Xing, L. M. Yerges-Armstrong, B. Yu, F. Zannad, J. H. Zhao, H. Hemingway, N. J. Samani, J. J. V. Mc Murray, J. Yang, P. M. Visscher, C. Newton-Cheh, A. Malarstig, H. Holm, S. A. Lubitz, N. Sattar, M. V. Holmes, T. P. Cappola, F. W. Asselbergs, A. D. Hingorani, K. Kuchenbaecker, P. T. Ellinor, C. C. Lang, K. Stefansson, J. G. Smith, R. S. Vasan, D. I. Swerdlow, R. T. Lumbers, Genome-wide association and Mendelian randomisation analysis provide insights into the pathogenesis of heart failure. *Nat. Commun.* **11**, 163 (2020).

79. J. P. Pirruccello, A. Bick, M. Wang, M. Chaffin, S. Friedman, J. Yao, X. Guo, B. A. Venkatesh, K. D. Taylor, W. S. Post, S. Rich, J. A. C. Lima, J. I. Rotter, A. Philippakis, S. A. Lubitz, P. T. Ellinor, A. V. Khera, S. Kathiresan, K. G. Aragam, Analysis of cardiac magnetic resonance imaging in 36,000 individuals yields genetic insights into dilated cardiomyopathy. *Nat. Commun.* **11**, 2254 (2020).

80. C. Giambartolomei, D. Vukcevic, E. E. Schadt, L. Franke, A. D. Hingorani, C. Wallace, V. Plagnol, Bayesian test for colocalisation between pairs of genetic association studies using summary statistics. *PLOS Genet.* **10**, e1004383 (2014).

81. C. Wallace, A more accurate method for colocalisation analysis allowing for multiple causal variants. *PLOS Genet.* **17**, e1009440 (2021).

82. M. Uhlen, L. Fagerberg, B. M. Hallstrom, C. Lindskog, P. Oksvold, A. Mardinoglu, A. Sivertsson, C. Kampf, E. Sjostedt, A. Asplund, I. Olsson, K. Edlund, E. Lundberg, S. Navani, C. A. Szigyarto, J. Odeberg, D. Djureinovic, J. O. Takanen, S. Hober, T. Alm, P.-H. Edqvist, H. Berling, H. Tegel, J. Mulder, J. Rockberg, P. Nilsson, J. M. Schwenk, M. Hamsten, K. von Feilitzen, M. Forsberg, L. Persson, F. Johansson, M. Zwahlen, G. von Heijne, J. Nielsen, F. Pontén, Proteomics. Tissue-based map of the human proteome. *Science* **347**, 1260419 (2015).
